# Physiological and flesh quality consequences of pre-mortem crowding stress in Atlantic mackerel (*Scomber scombrus*)

**DOI:** 10.1371/journal.pone.0228454

**Published:** 2020-02-13

**Authors:** Neil Anders, Ida Eide, Jørgen Lerfall, Bjørn Roth, Michael Breen

**Affiliations:** 1 Fish Capture Division, Institute of Marine Research (IMR), Bergen, Norway; 2 Department of Biological Sciences, University of Bergen, Bergen, Norway; 3 Department of Biotechnology and Food Science, Norwegian University of Science and Technology (NTNU), Trondheim, Norway; 4 Department of Processing Technology, Nofima, Stavanger, Norway; Radboud Universiteit, NETHERLANDS

## Abstract

In commercial wild capture pelagic fisheries it is common practice to crowd catches to high densities to allow efficient pumping onboard. Crowding during the final stages of purse seine capture for small pelagic species often results in intense and sustained behavioural escape responses. Such a response may trigger a shift in energy production from aerobic to anaerobic pathways and result in metabolic acid accumulation and exhaustion of intracellular reserves of ATP. Where there is insufficient time or opportunity to recover to physiological equilibrium before death, pre-mortem stress may be an important determinant of fillet quality, as has been shown for a variety of farmed fish species. However, there is currently a lack of knowledge related to the flesh quality implications of capture stress for wild captured species in European waters. Here we show that crowding results in a physiological stress response that has consequences for flesh quality in the wild captured species Atlantic mackerel (*Scomber scombrus*). Using small schools in tanks and aquaculture net pens in three separate experiments, we found crowding results in physiological changes in mackerel consistent with an acute stress response and anaerobic metabolism. Consequently, we found crowded fish had more acidic pre- and post-mortem muscle pH as well as indications of faster onset and strength of *rigor mortis* and increased cathepsin B & L activity. We examined fillet flesh quality after two and seven days of ice storage and found reduced green colouration, increased gaping (separation of muscle myotomes) and reduced textural firmness associated with fish which had been crowded. However, the effects on quality were dependant on experiment and/or storage time. These results indicate the potential of crowding capture stress to influence the flesh quality of an economically important species and may have important implications for the wild capture pelagic fishing industry.

## Introduction

External stimuli can be considered as stressors if they provoke regulatory mechanisms within an organism which attempt to establish an optimal physiological equilibrium for a given situation [1]. The collective function of these stress responses is to allow the animal to adaptively cope with challenges from its environment. However, when the magnitude of the stressor is extreme, the response may be rendered maladaptive and homeostasis may never be restored [2]. The same may occur when there is insufficient time available for the full recovery of the stress response back to baseline levels. Depending on practises, such time limited and extreme stressor situations may characterise the conditions animals experience prior to slaughter for human consumption. Consequently, pre-mortem stress has been shown to have flesh quality consequences for a range of animals, including both farmed (e.g. [3–6]) and wild captured (e.g. [7–10]) fish due to the intrinsic link between quality and physiological status at death [3,11].

During European purse seine fishing, entire schools consisting of several hundred tonnes of small pelagic fish can be caught. The net is then reduced in volume by hauling it aboard the catching vessel, which serves to crowd the catch into extremely high densities (perhaps to in excess of 200 kg/m^3^ [12]) to allow efficient pumping of the fish aboard. Duration of crowding is dependent on catch size, but pumping can take up to an hour or more to be completed [13]. Experimental observations suggests that purse seine crowding is associated with increases in physical activity levels [12,14,15], and anecdotal evidence from the fishery indicates that this activity can become intense during the final stages of capture. Behavioural activity at this stage is characterised by sustained and intense escape behaviour from the fish. Accordingly, crowding has been shown to initiate physiological responses in pelagic schooling fish that are of sufficient severity to cause mortality [16–19]. Considering that current practice involves pumping catches directly into refrigerated seawater (RSW) tanks, there is likely little opportunity for homeostatic recovery prior to death.

In such situations of intense physical activity from fish, the intracellular supply of oxygen is likely inadequate and shortages of adenosine triphosphate (ATP) can be expected due to the lower energetic yield of anaerobic respiration compared to aerobic pathways [20]. Concomitant lactate production and metabolic proton accumulation can also be expected [21]. This shift from aerobic to anaerobic catabolism can lead to metabolic acidosis and a faster onset and strength of post mortem muscle stiffening (*rigor mortis*) [22] due to the failure of muscular myosin and actin to separate owing to the exhaustion of adenosine triphosphate (ATP) reserves [23]. The effects of stress, rigor and acidosis are known to then act via a variety of interacting post-mortem physical, autolytic and metabolic pathways to influence ultimate flesh quality parameters such as texture [5,24,25], colour [26–28], incidence of residual blood in fillets [29,30], water holding capacity [7,31,32] and gaping (the undesirable separation of muscle myotomes) [32–34] in a wide variety of fish species.

Atlantic mackerel (*Scomber scrombrus*) are extensively targeted by the pelagic purse seine fishing industry throughout Europe [35]. Considering that catch value for this species depends on quality [36,37], knowledge related to the determinants of flesh quality may facilitate economic benefits for an important industry. Although studies indicate that a wide variety of post-mortem storage and handling procedures can affect quality [38], there is currently a lack of knowledge related to the quality implications of capture stress in this species.

Atlantic mackerel have previously been shown to be highly sensitive to capture related crowding [12,39] and other members of the *Scomber* genus display stress induced quality effects [40–45]. It is therefore possible that the current capture practise of extreme crowding impacts upon flesh quality. The objective of the current work was therefore to determine whether crowding stress can influence Atlantic mackerel quality. Based on the current understanding of the interaction between physiology and quality, it is reasonable to hypothesise that crowding will result in a stress response that will have negative consequences for flesh quality.

## Materials and methods

### Fish capture, husbandry and welfare

Wild mackerel were passively attracted using aquaculture feed pellets into a 12 x 12 x 12m aquaculture net pen (the “holding” net pen) at the Austevoll Research Facility (60°N) of the Institute of Marine Research, Norway during the summer and autumn of 2018. Fish were then transferred to one of two covered outdoor tanks (circular, 3m diameter, water depth ~ 1m) and allowed an acclimatisation period of at least 3 months prior to the start of experimentation. For transport to tanks, fish from the “holding pen” were first encouraged to begin feeding behaviour using feed pellets. Individual fish were then caught using barbless hooks (size: #1/0) and a handline, dehooked into 90 L seawater buckets lined with plastic bags (taking extreme care not to physically touch the fish [46]) and then “poured” into a randomly selected tank. Transport to tanks typically took < 10 mins. Tanks operated on a flow-through system, both supplied from the same natural deep sea-water source and had a photoperiod with 12 h reduced lighting and 12 h full lighting. Fish were intermittently fed aquaculture feed pellets up until 3 days before experimentation.

However, during experimental work it became apparent that collecting mackerel from the tanks for sampling was impossible to achieve without causing significant disturbance to the rest of the school. To minimise any disturbance related physiology effects, subsequent work was conducted in an “experimental” net pen (square, 5 x 5 x 5m) placed inside the “holding” net pen. This was stocked 3 days before the start of experimentation by encouraging fish to swim in voluntarily from the “holding” net pen using feed pellets. Rapid acclimation to the “experimental” pen was evident from the adoption of schooling and feeding behaviours consistent with behaviours observed in the “holding” pen. In net pens, fish foraged on natural food items.

All experimental protocols were prospectively authorised by the Norwegian animal welfare authority (Mattilsynet, FOTS licence IDs: 15113 and 19238) and undertaken by researchers with FELASA (Federation of European Laboratory Animal Science Associations) accredited laboratory animal science training. Welfare was monitored behaviourally on a daily basis before starting experimentation, with moribund or fish with severe skin lesions being removed and euthanised using a percussive blow to the head; a legal and humane killing method [47]. Housing with conspecifics ensured behavioural enrichment prior to experimentation. Stocking densities prior to treatment were non-limiting and animals could essentially adopt voluntary densities. Experimental design prospectively considered the 3R’s (Replacement, Reduction and Refinement). There was no practical alternative to the use of live animals. The number of animals used per experiment (n ≤ 25) was the minimum required to obtain a reasonable threshold of certainty for statistical validation. The total number of animals used was 52. The exposure of subjects to experimental treatment did not exceed 60 minutes. No anaesthesia was used as to do so would compromise the experimental objectives. The pre-defined humane endpoint was a sustained loss of schooling behaviour, with “sustained” defined as continuing for sufficient duration so that fish show signs of fatigue manifested as a loss of equilibrium in individuals. In such cases, fish were euthanised immediately using a percussive blow to head or by anesthetic overdose (MS-222 at 500 mg/L). No fish reached this endpoint criteria and no fish died before meeting the endpoint criteria. Instead, all fish used in the experiments were killed by a percussive blow to the head to allow blood and tissue sampling.

### Treatment

Three experiments were conducted to determine the response of mackerel to crowding; two in tanks (Experiments E19 and E20) and one in the experimental net pen (Table 1). The experimental design consisted of pre- and post-treatment sampling on separate groups of fish for each experiment. Before commencing any crowding treatment in each of the experiments, ~ 50% of fish were removed to act as control samples. To achieve this in tanks, the volume of water was reduced to ~ 20 cm depth and randomly selected fish were caught with a hand net; control fish in the net pen were encouraged to begin feeding behaviour using feed pellets and then caught by hook and handline. Although this technique was initially successful, fish in the “experimental” net pen soon became reluctant to bite the handline hook. Therefore, 6 of the 10 control fish for the net pen experiment were collected from the “holding” net pen using the same method. Once hooked, fish were immediately removed from the water.

**Table 1:** Experimental design. Detail of the different experiments and associated sample sizes used to assess physiological and flesh quality responses of mackerel exposed to crowding.

			No. of fish			Ambient conditions during crowding		
Experiment	Experimental arena	Date	Control	Crowded	Total	Water temperature (°C)	Mean oxygen saturation (%)	Minimum oxygen saturation (%)
E19	Aquarium tank	17/01/2019	7	6	13	Data missing*	Data missing*	Data missing*
E20	Aquarium tank	17/01/2019	10	9	19	7.5	83	74
Net pen	Aquaculture net pen	28/02/2019	10	10	20	6.2	98	83

* CTDO failure

Following collection of control fish, crowding treatment immediately commenced. For this, all remaining fish were enclosed inside a “crowding device”, constructed from commercial purse seine netting (mesh size: 15 mm). Crowding devices were placed inside the experimental arena and were conical in shape, closed at the bottom and with an opening diameter at the top of ~ 1m (tank experiments) or ~ 2m (net pens). Both crowding devices were ~ 1m deep. In tanks, fish were caught individually by hand net and placed inside the crowding device. Water depth remained at ~ 20cm during treatment. Efforts were made to minimise the “chasing” of fish during this procedure. In the net pen experiment, the crowding device was first placed at the bottom of the pen. The volume of the whole pen was then reduced by lifting it vertically up in the water, causing the schooling mackerel to gather inside the crowding device. The crowding device was then lifted further vertically from the water, trapping most of the school inside. Fish which were not inside the device after this process where caught by hand net and placed inside. Crowding density could be controlled by lifting the crowding devices vertically from the water, thereby reducing the available swimming space. It was not possible to accurately determine density during treatment. However, fish were crowded beyond voluntary swimming densities and to the point that normal swimming behaviour was inhibited, with numerous and regular contact between fish and with netting but always ensuring that the majority of fish were below the water line. It is however likely that the crowding densities applied were not exactly the same between the different experiments. Single fish were then randomly collected from the crowding devices by hand (the tank experiments) or hand net (the net pen experiment). Collection of fish continued until the net was empty. This took 34, 59 and 60 minutes for Experiment E19, E20 and the net pen respectively.

### Responses

Due to logistical constraints, transportation issues and the use of fish from Experiment E20 for a separate freezing quality experiment (not reported here), the responses measured between the experiments were not identical. Refer to S1 Table for detail of which responses were measured during which experiment. Detail regarding specific procedures are included below.

#### Blood physiology and muscle pH—Sample collection

Once landed, fish were immediately euthanised using a percussive blow to the head and marked with a unique identifying T-Bar anchor tag in the gill operculum. Blood (typically ~ 1.5 – 3mL) was collected from caudal vein puncture as soon as possible after euthanisation (typically within 1 minute) using 5ml syringes fitted with 21G needles. Syringes were prior treated with 10% EDTA (ethylenediaminetetraacetic acid) solution as an anticoagulant. A drop of blood was then subsampled onto test strips for immediate analysis of whole blood lactate and glucose, using the Lactate Pro 2 (Arkray Inc., Kyoto, Japan) and Contour Next One (Ascensia, Basel, Switzerland) point-of-care (POC) devices respectively (please refer to S1 Validation for further detail of these devices). Blood was then chilled on ice for up to a maximum of ~ 4 hours for later processing. Although immediate processing may have been preferable so as to reduce any post mortem changes in blood parameters [48], this was not practically feasible. The refrigeration of whole blood samples is preferable to room temperature storage [49] and is common practice in field studies of animal physiology. Fork length and total weight was then measured, before cutting two muscle samples of ~ 1cm^3^ each from the dorsal side of the left fillet immediately posterior to the gill operculum for analysis of muscle physiology (results not presented here). Muscle pH was then recorded using a puncture electrode coupled to a calibrated SevenGo Duo pH meter (Mettler Toledo, Greifensee, Switzerland) inserted into the muscle of the dorsal loin in the same region from which the muscle samples were collected from. Fish were then immediately stored whole on ice in expanded polystyrene (EPS) boxes.

#### Blood physiology—Sample preparation and analytical methods

A subsample of ~0.6 μL chilled whole blood was placed in a heparinised capillary tube for haematocrit quantification by centrifuging at room temperature for 5 mins at 16,000 *g* using a Haematokrit 2010 (Hettich Zentrifugen, Tuttlingen, Germany). Remaining blood was then centrifuged at room temperature for 5 mins at 2000 *g* using either a MiniStar Silverline (VWR International, Radnor, USA) or a Spectrafuge mini centrifuge (LabNet International, Edison, USA) and resulting plasma frozen at -80°C. In the laboratory, defrosted plasma was analysed for concentrations of glucose, potassium, sodium and chloride ions as well as osmolality and pH using an ABL90 FLEX (Radiometer Medical, Brønshøj, Denmark) blood gas analyser. The ABL90 was also used to analyse plasma lactate from the net pen experiment. Otherwise, plasma lactate was analysed by diluting the sample by a factor of 4 (using 9 mg/L NaCl in distilled water) and quantified using a MAXMAT PL biomedical analyser (MAXMAT SA, Montpellier, France) with a lactate assay kit (Medicon Hellas, Gerakas, Greece). A MAXMAT PL was also used to quantify alanine transaminase (ALT), aspartate transaminase (ASP), phosphate (all using MAXMAT supplied assay kits) and triglyceride concentrations (Medicon Hellas assay kit). Plasma cortisol concentration was quantified using a Sunrise (Tecan Group, Männedorf, Switzerland) microplate reader with an ELISA essay kit (IBL International, Hamburg, Germany).

#### Post-mortem rigor and pH development

Rigor status and muscle pH were examined at seven post-mortem intervals (0, 3, 6, 12, 18, 24 and 43 hours after death) in all fish from Experiment E19. pH was measured in the same way and at the same location as for muscle pH immediately post-mortem. Changes in muscle stiffness over time was determined using Cutting's tail drop method [50], by measuring the vertical drop (cm) of the tail when half of *Rigor angle*° = *tan*^−1^(*a/b*) the fish fork length is placed (right fillet on top) on the edge of a table. Rigor angle was calculated as:, where *a* = half the fish fork length (cm) and *b* = the vertical drop of the tail, with a rigor angle = 0° indicating extreme muscle stiffness (no tail drop) and 90° indicating the complete absence of muscle stiffness (vertical tail drop, unlikely to occur due to skeletal support). In between assessments, fish were stored whole on ice in EPS boxes.

#### Quality—Storage times and conditions

Assessment of quality was undertaken two days after death and/or seven days after death, dependant on experiment. In the mackerel fishery, two days is the typical time for catches to arrive at the processing factory while seven days corresponds to the probable latest point a mackerel would be sold fresh. All fish from Experiments E19 and E20 were first stored whole on ice in EPS boxes for two days, then filleted by hand and various quality metrics collected (data collected at this time point is hereafter referred to as “Day 2”). In the case of Experiment E19, fillets were then vacuum packed and stored on ice in a refrigeration unit (temperature: 4°C) and examined for quality after five days (hereafter referred to as “Day 7”). For reasons related to transport, fish from the net pen experiment were first gutted. They were then stored on ice in a refrigeration unit (temperature: 4°C) for a total of seven days, then filleted by hand and quality assessed (*i*.*e*. assessed on “Day 7”). After quality assessment, fillets from the net pen experiment were frozen at -80°C for later analysis of cathepsin activity (see below). Sex and the presence/absence of any pre-mortem skin injuries and the weight of individual fillets was determined at the point of filleting in all experiments.

#### Quality–sample analysis

Colour of the fillets was determined digitally using a DigiEye system (VeriVide Ltd., Leicester, UK). Fillets were placed in a light box with standardized illuminant (D65/10°, simulating outdoor daylight) and photographed using a colour calibrated digital camera (Nikon D80 with a 35 mm lens, Nikon, Tokyo, Japan). Using the associated DigiPix software, we selected an area spanning from the dorsal to ventral side of the right-hand fillet immediately posterior to the pectoral fin to quantify skin colour. The width of the area was approximately equal to the length of the pectoral fin. To quantify flesh colour, a randomly selected area at the approximate midpoint of the dorsal side of both fillets was used. As large an area as possible was selected, making sure not to include any instances of gaping or blood spotting. Colour was quantified within these selected areas in CIELAB and CIELCh colour space in terms of lightness (L*, 0 = black, 100 = white), red / green intensity (a*, < 0 = green, > 0 = red), yellow / blue intensity (b*, < 0 = blue, > 0 = yellow), saturation (“chroma”, calculated as C* = √(a*2 + b*2) where 0 = unsaturated and 100 = fully saturated,) and hue angle (h°, calculated as h° = tan-1(b*/a*) where 0° = reddish hue and 90° = yellowish hue) [5,51]. Fillets were visually assessed blind by a single researcher and assigned a score to describe the degree of gaping and blood spotting (Table 2, based on [13,52]).

**Table 2:** Scoring system used for assessment of gaping and blood spotting. Gaping score methodology based on Grimsmo [52]. Blood spotting scoring adapted from Digre *et al*. [13].

Metric	Score	Description
Gaping	0	No gaping
	1	A few small splits (1–5)
	2	Numerous small splits (6–10)
	3	Many small splits (>10) or a few large splits (1–5)
	4	Numerous large splits (6–10)
	5	Extreme gaping–numerous large splits (>10) and flesh detaches easily
Blood spotting	0	No visual blood spots
	1	Some small blood spots (<5)
	2	Large blood spots or several small (>5)

Fillet drip loss (DL) was determined from right-hand fillets over a period of 5 days of ice storage, using the following formula: *DL* = (*W*_*Day* 2_−*W*_*Day* 7_)/*W*_*Day* 2_×100, where *W*_*Day* 2_ and *W*_*Day* 7_ is fillet weight on Day 2 and Day 7 respectively. To assess water holding capacity (WHC), two muscle tissue samples of ~ 5 g were cut from the left-hand fillet and placed into liquid separating cylinders (Part No. 4750, Hettich Lab Technology, Tuttlingen, Germany). Samples were then centrifuged at 530 *g* for 15 min at 4°C using a Rotina 420 R (Hettich Lab Technology, Tuttlingen, Germany) with a free swing rotor. The separated liquid content during centrifuging was then determined gravimetrically. WHC was calculated as: *WHC* = *W*_0_−Δ*W*/*W*_0_×100, where *W*_0_ = *V*_0_/(*V*_0_+*D*_0_)×100 and Δ*W* = Δ*V*_0_/(*V*_0_+*D*_0_)×100, with *V*_0_ = the water content of the muscle, *D*_0_ = dry mass of the muscle and Δ*V*_0_ = the weight of the liquid separated during centrifuging [53]. *D*_0_ and thereby *V*_0_ were determined gravimetrically by drying two additional muscle samples for 20–22 hours at 105°C.

Flesh texture was determined using a TA-XT2 textural analyzer (Stable Micro Systems, Surrey, UK) equipped with a 25 kg load cell and a flat-ended cylinder probe (20 mm diameter, type P/1SP). The maximum force required to compress the fillet to 80% of its height was recorded by the associated Texture Exponent software (Stable Micro Systems) as a measure of fillet firmness. Firmness was analysed at two immediately adjacent locations per fillet, situated on the flesh side and at the approximate midpoint of the most anterior end of the fillet.

#### Cathepsin B & L activity–sample preparation and analytical methods

Increases in cathepsin B & L (intracellular proteases typically located within the lysosome) activity has previously been associated with autolysis and post-mortem muscle softening in a variety of teleost fish species [54], including the *Scomber* genus [55]. A pre-trial was first performed for the optimization of the procedure for Atlantic mackerel, with a pH of 6.0 for the phosphate buffer and an incubation temperature of 50°C showing the most stable results. Using fish from the net pen experiment, two muscle samples of ~ 1 g each were cut from defrosted fillets and homogenized with 5 ml of phosphate buffer (consisting of 3.38 mM Na_2_HPO_4_, 15 mM NaH_2_PO_4_, 0.25 M sucrose, 1mM EDTA and 100 mM NaCl) for 40 seconds at 13,000 RPM by an Ultra Turrax T25 disperser (IKA-Werke GmbH & Co, Staufen, Germany). Samples were chilled on ice during homogenization. Samples were then centrifuged at 15,000 *g* in an Avanti JXN-26 centrifuge (Beckman Coulter, Brea, USA) for 20 minutes at 4°C. 15 μl of the resulting supernatant was added to 135 μl of activation buffer (340 mM CH_3_COONa, 60 mM CH_3_COOH, 4 mM EDTA, 0.1% Brij 35, 0.8 M Dithiothreitol C_4_H_10_O_2_S_2_), mixed and placed in an incubator at 50°C for 10 minutes. Thereafter, 100 μl of 12.5 μM substrate solution (10 mM Z-Phe-Arg-Nmec/AMC in 1 ml dimethyl sulfoxide) was added and incubated at 50°C for a further 10 minutes. Finally, 1 ml of stop buffer (100 mM NaOH, 30 mM CH_3_COONa, 70 mM CH_3_COOH, 100 mM CH_2_ClCOOH) was added. 250 μl of the prepared sample was added to a microtiter plate together with a standard dilution series (10 mM 7-amino-4-methyl coumarin) and three blank samples. Samples were then analyzed by a fluorometer (Synergy 2, BioTek Instruments Inc., USA) with excitation and emission wavelengths of 355nm and 460nm respectively being recorded.

### Statistical analysis

Data exploration was conducted following the procedures described by Zuur [56]. Two obviously erroneous outliers (one from the cathepsin dataset and one from the texture dataset) were identified using Cleveland dot plots and were removed. Samples above or below the limit of detection (LOD) of the analytical machine were assigned either the value of the upper or lower limit as quoted by the manufacturer. This was 0.07 mM for phosphate (2 samples were <LOD), 0.04 mM for triglycerides (1 sample was <LOD) and 800 ng/ml for cortisol (11 samples were >LOD).

All statistical analysis was undertaken using R version 3.4.2 [57], with the level of significance set at 0.05. The biometric and drip loss data was modelled using simple linear regression (ANOVA) or binomial generalized linear modelling (GLM) with either *F* or Chi-squared (χ2) testing respectively to determine the significance of covariate terms. We modelled the blood/tissue physiology and skin colour datasets using Generalized Least Squares (GLS) regression in the “nlme” R package [58], to allow us to incorporate identifiable residual dependency into the models [59]. The applied variance structure (either VarIdent, VarExp or VarPower) was selected using Akaike information criterion (AIC) and likelihood ratio testing (LRT). For datasets where more than one replicate value was obtained from the same fish (texture, flesh colour, cathepsin and WHC), we applied linear mixed effects models (LMM) in the “nlme” package [58] with random effects at the level of individual fish to avoid pseudo-replication [60]. Again, we incorporated any identified residual dependency using variance structures. For data which were ordinally distributed (i.e. the blood spotting and gaping scores), we fitted Cumulative Link Mixed Models (CLMM) with logit link functions from the “ordinal” R package [61] to determine how covariates influenced the probability of obtaining a given scores. We also included random effects at the level of individual fish in the CLMM’s and determined significance of model terms using LRT. We obtained predicted mean scores from CLMM’s using the “lsmeans” package [62]. The development of post-mortem pH over time showed a clear non-linear relationship and was consequently modelled using Generalized Additive Mixed Modelling (GAMM) [59] in the “mgcv” R package [63]. We used a gaussian (link function = identity) error structure and included individual fish level random effects. Post-mortem rigor angle was similarly modelled, but with a binomial (link function = logit) error structure and as a Generalized Additive Model (GAM) as AIC testing showed the inclusion of random effects did not improve model fit. In addition, we fitted a generalised linear mixed model (GLMM) to determine rigor angle differences between groups during the onset of rigor. Correlations between variables were investigated by using Pearson’s correlation (for normally distributed data) or Spearman’s rank correlation (non-parametric data). Where appropriate, we examined plots of residuals against fitted values, model covariates and expected distribution (Q-Q plots) to ensure model assumptions of residual independence and normality were not violated.

Where sampling effort allowed, maximal models consisting of an interaction between the covariates of “Treatment” (categorical: “control” or “crowded”) and “Experiment” (categorical: “Tank E19”, “Tank E20” or “net-pen”) were first fitted. For the texture dataset, the additional covariate of fillet weight (continuous) to account for potential differences in textural properties between fillets of different sizes [64] was included. As well as the interaction models, a separate main effects only model was fitted and significance or AIC testing was used to determine the most parsimonious. Due to collinearity between “Day” (days after death; categorical: “Day 2” or “Day 7”) and “Experiment”, data from different days was modelled separately. In the case of the GAM’s, “Time” (continuous: hours post mortem) was included as an isotropic smoothing function (type: thin-plate regression spline [59]) with an interactive effect of “Treatment”.

## Results

### Biometrics

A total of 52 individual mackerel (27 female, 25 male) were used throughout the three experiments. Overall mean (± SD) fork length, total weight and condition index (100 x [weight / body length^3^]) was 38.33 ± 2.04 cm, 553.06 ± 108.46g and 0.98 ± 0.13 respectively. Mean length (E19 = 38.65 ± 2.54 cm; E20 = 38.08 ± 1.92 cm; net pen = 38.35 ± 1.87 cm) and weight (E19 = 547.53 ± 111.11 g; E20 = 513.05 ± 115.26 g; net pen = 594.65 ± 88.05 g) were not significantly different between the three experiments (ANOVA, F_(2,49)_ = 0.29, p = 0.74 and F_(2,49)_ = 2.99, p = 0.06 respectively) but fish condition (F_(2,49)_ = 7.64, p < 0.01) tended to be lower in the tank experiments (E19 = 0.94 ± 0.09; E20 = 0.92 ± 0.11) than in the net pen (1.06 ± 0.14). There were no significant differences in length, weight and condition between control and treatment groups in each experiment (ANOVA, p > 0.05 in all cases). 75% of tank fish had obvious pre-mortem skin injuries of some kind, while this effect was not seen at all for net pen fish. There was no significant difference in the incidence of injuries between control and crowded fish from within each tank experiment (binomial GLM, p > 0.05 in both cases), suggesting the injuries did not arise due to treatment.

### Blood physiology and initial muscle pH

**Fig 1:**
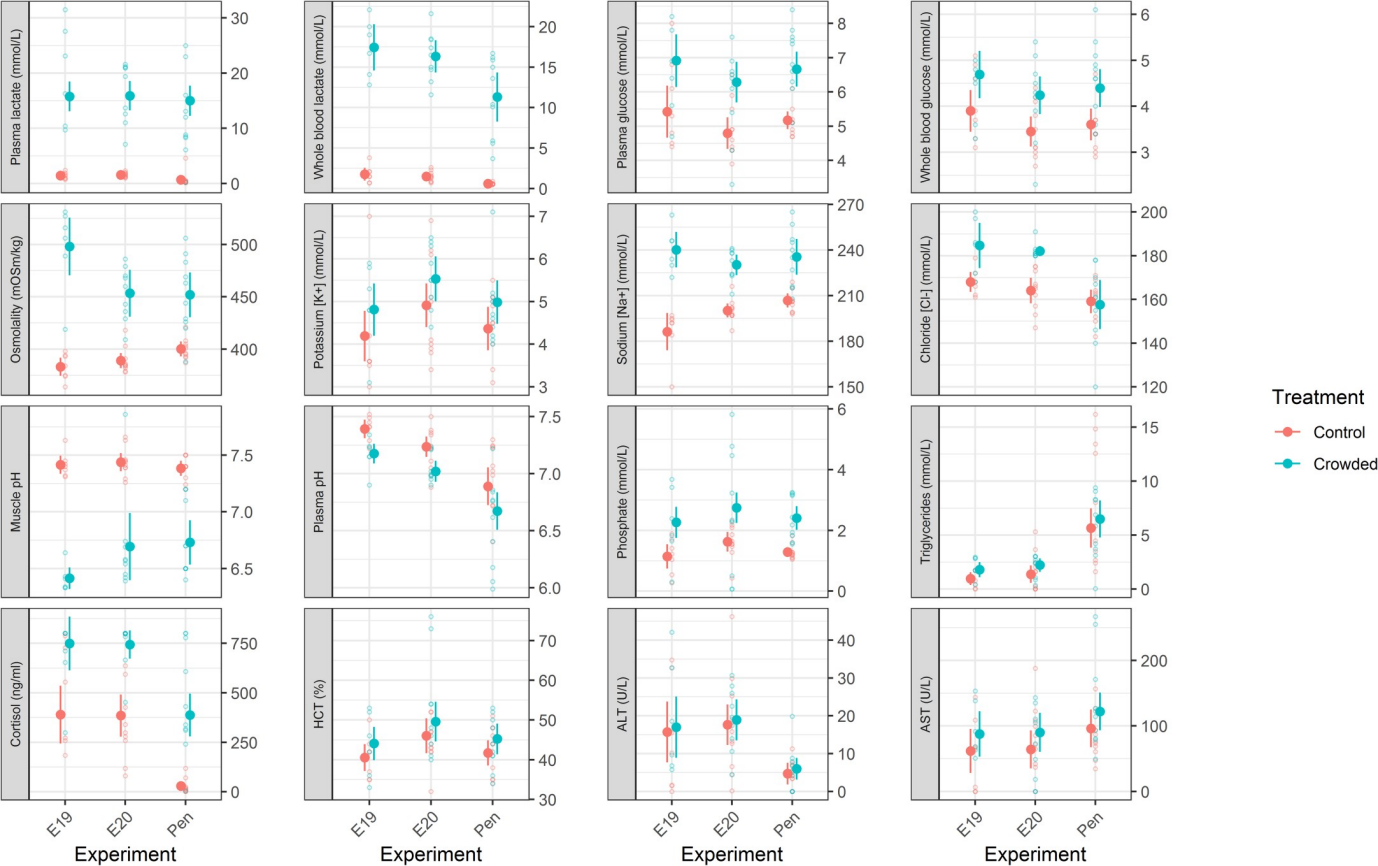
Blood and muscle physiology responses to crowding in Atlantic mackerel. Model predicted mean control and crowded values are shown as filled circles, with associated 95% confidence intervals as whiskers. The underlying dataset is shown as open circles. Data points are coloured according to treatment. HCT refers to haematocrit, ALT to alanine transaminase and ASP to aspartate transaminase.

The main effect models fitted to the blood and tissue physiological data showed significant effects (p < 0.05 in all cases) of “treatment” for all parameters apart from haematocrit (p = 0.09), ALT (p = 0.59) and AST (p = 0.09). There were some significant interactive effects with “experiment” (Table 3). The significance of the “experiment” term in a number of the models indicated differences in either control and/or crowded parameter values between the different experiments. In such cases, the net-pen experiment values tended to be different from the tank results, which were more similar to one another (Fig 1).

**Table 3:** Blood and muscle physiology statistical results. Statistical test results and associated significance of the interaction and main effects of “treatment” (either control or crowded) and “experiment” (either E19, E20 or net pen) on various Atlantic mackerel blood and muscle physiological parameters. Models were fitted using Generalized Least Squares (GLS) regression with a variety of variance structures, as indicated.

			Main effects model						Interaction model		
			X_1_ (“Treatment”)			X_2_ (“Experiment”)			X_3_ (X_1_ × X_2_ interaction)		
Parameter	Variance structure	n	df	*F*-value	*p*	df	*F*-value	*p*	df	*F*-value	*p*
Cortisol	VarIdent	52	1	351.596	<0.001	2	24.498	<0.001	2	1.5084	0.232
Lactate (whole blood)	VarIdent	52	1	400.161	<0.001	2	15.443	<0.001	2	3.175	0.051
Lactate (plasma)	VarIdent	52	1	108.281	<0.001	2	2.097	0.134	2	1.387	0.260
Haematocrit	VarExp	52	1	3.019	0.089	2	2.192	0.123	2	0.715	0.495
pH (plasma)	VarIdent	52	1	20.46	<0.001	2	17.70	<0.001	2	1.25	0.297
pH (muscle)	VarIdent	52	1	297.92	<0.001	2	0.93	0.399	2	4.46	0.017
Osmolality	VarIdent	52	1	72.31	<0.001	2	3.20	0.049	2	5.99	0.005
Glucose (whole blood)	VarPower	52	1	14.274	<0.001	2	1.406	0.255	2	1.543	0.225
Glucose (plasma)	VarIdent	52	1	33.110	<0.001	2	1.477	0.239	2	1.347	0.270
Potassium ions	none	52	1	5.068	0.029	2	2.457	0.096	2	0.357	0.702
Sodium ions	VarIdent	52	1	90.192	<0.001	2	3.018	0.058	2	3.417	0.041
Chloride ions	VarIdent	52	1	82.99	<0.01	2	6.01	0.005	2	3.87	0.028
Alanine transaminase (ALT)	VarIdent	52	1	0.301	0.586	2	13.035	<0.001	2	0.154	0.858
Aspartate transaminase (AST)	none	52	1	3.009	0.089	2	2.152	0.127	2	1.791	0.178
Phosphate	VarIdent	52	1	28.159	<0.001	2	2.194	0.123	2	0.315	0.969
Triglycerides	VarIdent	51	1	9.751	0.003	2	13.022	<0.001	2	1.649	0.204

Plasma ion (chloride, potassium, sodium, osmolality) concentrations were generally elevated by crowding as were whole blood and plasma glucose (Fig 1). Whole blood and plasma lactate increased substantially (by a mean factor of 12 and 13 respectively) in response to crowding (Fig 1) and correlated significantly with the observed reductions (Fig 1) in muscle pH (Spearman's rank correlation of lactate and muscle pH; whole blood lactate: ρ = -0.75, p < 0.001 and plasma lactate: ρ = -0.73, p < 0.001, S1 Results). Plasma pH also became more acidic in response to crowding, while cortisol levels increased by a mean factor of 2 in the tank experiments and by a factor of 14 in the net-pen experiment (Fig 1). Phosphate and triglyceride levels also increased. There was good correlation between whole blood values as measured by the POC meters with plasma values obtained in the laboratory for lactate (Spearman's rank correlation, ρ = 0.87, p < 0.001) and glucose (ρ = 0.81, p < 0.001, S1 Validation).

### Rigor development and post-mortem pH

**Fig 2:**
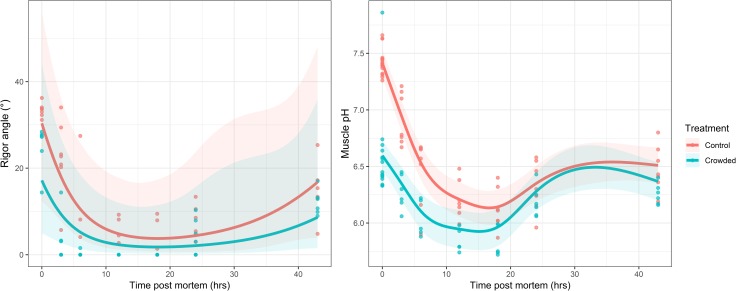
**Post-mortem rigor angle development (left panel) and muscle pH (right panel) response to crowding in Atlantic mackerel.** The lines indicate the fitted relationship, with the 95% confidence intervals shown as shaded areas. The underlying dataset is shown as filled circles. Data points and lines are coloured according to treatment.

The GAM’s fitted to the rigor angle (n = 88) and muscle pH (n = 107) data collected from Experiment E19 suggested that while pH values differed significantly across time (Time:Treatment interaction smoother: *F* = 25.76, estimated df = 3.54, p < 0.001), rigor angle did not (Time smoother: *χ*^2^ = 5.83, estimated df = 2.22, p = 0.09). However, the fitted relationship (Fig 2) would suggest mackerel reached maximum stiffness ~18 hours post-mortem, which began to resolve ~3 hours later (Fig 2, left panel) which approximately corresponded to the lowest post-mortem pH values (Fig 2, right panel). Fish with lower initial muscle pH also tended to obtain maximum rigor stiffness at a faster rate (Spearman's rank correlation: ρ = 0.70, p < 0.01, S1 Results).

Crowded fish were noticeably stiffer in the immediate post-mortem period than control fish, tended to have faster rigor onset, obtained a higher degree of stiffness and took take longer to resolve rigor (Fig 2, left panel). Prior to reaching the point of maximum stiffness, there was a significant interactive effect of time and treatment (GLMM, LRT = 3.75, df = 1, p = 0.05). However, the overall time course of rigor angle in crowded fish was not significantly different from the control group (*χ*^2^ = 1.05, df = 1, p = 0.31). This is in contrast to changes in post-mortem muscle pH over time (Fig 2, right panel), where the fitted GAMM indicated highly significant differences between the crowded and control groups (*F* = 23.0, df = 1, p < 0.001). Crowded fish became more acidic (mean pH minimum for crowded = 5.92; control = 6.13) at a faster rate than the control group (time to pH minimum for crowded = ~15 hours post-mortem; control = ~17 hours, Fig 2, right panel). Towards the end of the observation period, the pH of both groups became less acidic and converged (Fig 2, right panel).

### Colour

#### Skin colour

We examined skin colour after two days of ice storage from the tank experiments (Table 4). Although there were significant differences in some colour parameters between the two tank experiments, no significant effect of crowding or interaction effect with experiment (p > 0.05 in all cases) on mackerel skin colour was detected (Table 5).

**Table 4:** Colour parameter values in response to crowding in Atlantic mackerel skin and flesh. Colour was determined photometrically either two (“Day 2”) or seven (“Day 7”) days after death, in terms of CIELAB colour space. Values indicate mean ± 95% confidence intervals.

			Experiment					
			E19		E20		Pen	
Location	Colour parameter	Day	Control	Crowded	Control	Crowded	Control	Crowded
Skin	Colour lightness (L*)	Day 2	48.064 ± 0.779	47.225 ± 0.388	48.806 ± 1.028	50.167 ± 1.828	-	-
	Red / green axis (a*)		-8.419 ± 0.349	-8.093 ± 0.579	-7.795 ± 1.234	-8.190 ± 0.754	-	-
	Yellow / blue axis (b*)		15.111 ± 1.493	16.350 ± 1.196	14.187 ± 1.942	11.859 ± 2.896	-	-
	Chroma (C*)		17.344 ± 1.137	18.278 ± 0.859	16.431 ± 1.380	14.703 ± 2.215	-	-
	Hue angle (h°)		119.473 ± 3.574	116.520 ± 3.128	119.814 ± 6.478	126.928 ± 7.502	-	-
Flesh	Colour lightness (L*)	Day 2	52.415 ± 1.413	50.910 ± 1.584	50.914 ± 0.870	52.298 ± 1.102	-	-
	Red / green axis (a*)		-5.198 ± 0.455	-2.232 ± 1.170	-4.560 ± 0.490	-4.015 ± 1.027	-	-
	Yellow / blue axis (b*)		6.009 ± 1.771	8.603 ± 3.125	8.269 ± 1.468	5.524 ± 2.124	-	-
	Chroma (C*)		8.375 ± 1.127	9.923 ± 2.078	9.714 ± 1.158	7.853 ± 1.470	-	-
	Hue angle (h°)		136.907 ± 13.027	117.890 ± 22.801	122.323 ± 6.866	137.667 ± 14.805	-	-
	Colour lightness (L*)	Day 7	51.417 ± 1.675	51.097 ± 1.693	-	-	52.565 ± 0.897	53.971 ± 1.035
	Red / green axis (a*)		-4.584 ± 0.982	-1.442 ± 1.673	-	-	-4.375 ± 0.494	-3.276 ± 0.619
	Yellow / blue axis (b*)		8.257 ± 2.551	9.162 ± 4.006	-	-	6.439 ± 1.477	3.811 ± 2.134
	Chroma (C*)		9.794 ± 1.776	9.922 ± 3.050	-	-	8.117 ± 1.210	5.927 ± 1.644
	Hue angle (h°)		122.324 ± 12.678	108.973 ± 24.551	-	-	129.905 ± 8.210	143.079 ± 13.968

**Table 5:** Skin and flesh colour statistical results. Statistical test results and associated significance of the interactive and main effect of “treatment” (either control or crowded) and “experiment” (either E19, E20 or net pen) on mackerel skin and flesh colour parameters. Colour was assessed after two (“Day 2”) and/or seven days (“Day 7”) of ice storage. Skin models were fitted using Generalized Least Squares (GLS) regression. Flesh models were fitted using linear mixed effects models (LMM). The applied variance structure is indicated.

				Main effects model						Interaction model		
				X_1_ (“Treatment”)			X_2_ (“Experiment”)			X_3_ (X_1_ × X_2_ interaction)		
Location	Colour parameter	Variance structure	Day	df	LRT	*p*	df	LRT	*p*	df	LRT	*p*
Skin	Colour lightness (L*)	VarPower	Day 2	1	1.68	0.205	1	11.69	0.002	1	3.23	0.083
	Red / green axis (a*)	VarIdent		1	0.393	0.536	1	0.478	0.495	1	0.782	0.384
	Yellow / blue axis (b*)	VarIdent		1	0.1904	0.666	1	6.193	0.019	1	3.141	0.087
	Chroma (C*)	VarExp		1	0.215	0.647	1	8.617	0.007	1	2.957	0.097
	Hue angle (h°)	VarIdent		1	0.237	0.629	1	3.019	0.093	1	3.229	0.083
Flesh	Colour lightness (L*)	none	Day 2	1	0.007	0.798	1	0.032	0.856	1	3.042	0.081
	Red / green axis (a*)	VarIdent		1	5.755	0.016	1	0.650	0.420	1	4.195	0.041
	Yellow / blue axis (b*)	none		1	0.159	0.690	1	0.024	0.876	1	3.428	0.064
	Chroma (C*)	none		1	0.235	0.628	1	0.063	0.803	1	3.017	0.082
	Hue angle (h°)	VarIdent		1	0.023	0.879	1	0.030	0.863	1	3.052	0.081
	Colour lightness (L*)	none	Day 7	1	1.076	0.299	1	6.134	0.013	1	1.348	0.246
	Red / green axis (a*)	none		1	11.928	<0.001	1	1.966	0.161	1	4.247	0.039
	Yellow / blue axis (b*)	none		1	0.874	0.349	1	5.451	0.019	1	1.589	0.207
	Chroma (C*)	none		1	1.559	0.212	1	5.883	0.015	1	1.160	0.281
	Hue angle (h°)	VarIdent		1	0.159	0.689	1	4.851	0.028	1	2.508	0.113

#### Flesh colour

The fitted LMM models detected significant main effects of crowding on both Day 2 and Day 7 (p < 0.01 in both cases) on the expression of red/green (a*) colour axis, both of which interacted with “Experiment” (Table 5). Crowded fish tended to be less green (40% mean increase in a*) than their respective controls, and greenness also decreased for both control and crowded fish (11 and 35% increase in a* respectively) from Experiment E19 during storage (Table 4). The decrease in greenness was more pronounced for Experiment E19 than for Experiment E20 or the net pen experiment (Table 4). Otherwise, crowding did not significantly affect flesh colour properties (main effect models, p > 0.05 in all cases, Table 5) and did not interact significantly with experiment (interaction models, p > 0.05 in all cases, Table 5). More greenish fillets tended to have significantly lower firmness on Day 2 (Spearman's rank correlation: ρ = -0.84, p < 0.001, S1 Results). Softer fillets on Day 7 also tended to have less greenish colouration (ρ = -0.35, p = 0.04) as well as reduced colour lightness (Pearson’s correlation: *r* = 0.35, p = 0.04), more yellowness (*r* = -0.45, p = < 0.01) and higher colour saturation (C*) (*r* = -0.46, p = < 0.01), although the correlation between these variables was weak (S1 Results).

### Blood spotting and gaping

The mean (± SD) blood spotting score for fillets stored for two (n = 64) and seven days (n = 53) was 0.34 ± 0.67 and 0.169 ± 0.43 respectively and the majority of fillets contained no visible blood spots (77% and 85% of fillets obtained a score = 0 at Day 2 and Day 7 respectively). This was supported by the CLMM’s fitted to the data, which predicted the most probable outcome to be a score = 0 across all experiments on both Day 2 and Day 7 of storage. Furthermore, there was no significant effect of crowding on blood spotting scores on either Day 2 (LRT = 0.71, df = 1, p = 0.39) or Day 7 (LRT = -1.64, df = 1, p = 0.99, S2 Results).

**Fig 3:**
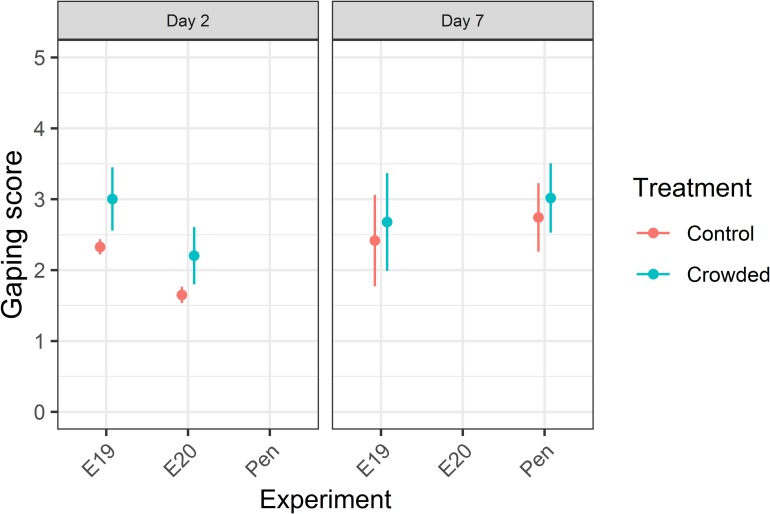
Post-mortem gaping response to crowding in Atlantic mackerel fillets. Gaping scores were obtained either two (“Day 2”) or seven (“Day 7”) days after death. Model predicted mean values are shown as filled circles for the different experiments, with associated 95% confidence intervals as whiskers. Data points are coloured according to treatment.

The CLMM’s fitted to the gaping data indicated the most probable score for either a control or crowded fish stored for seven days (n = 53) was 1; for a two day (n = 64) stored fish it was 0. The CLMM also demonstrated a significant effect of crowding on the expression of gaping on Day 2 (LRT = 4.76, df = 1, p = 0.03) but not on Day 7 (LRT = 0.71, df = 1, p = 0.40). Model predicted values showed that at Day 2, crowded fish tended to have slightly higher and more variable mean gaping scores than control fish, with values tending to converge by Day 7 (Fig 3). Two day stored fillets with higher gaping scores tended to have had lower initial muscle pH (Pearson’s correlation: *r* = -0.44, p = 0.01) and shorter times to obtain their maximum rigor stiffness (*r* = -0.60, p = 0.03) but gaping scores were not significantly correlated with fillet firmness (*r* = -0.37, p = 0.21) (S1 Results). After seven days of storage, gaping scores no longer significantly correlated with either initial muscle pH (*r* = -0.27, p = 0.13) or maximum rigor development time (*r* = -0.48, p = 0.08) (S1 Results).

### Drip loss and water holding capacity

Mean (± SD) percentage drip loss for fillets from Experiment E19 stored for 5 days (from Day 2 till Day 7) was 2.75 ± 0.95%, and was not significantly influenced by crowding (ANOVA, F_(1,11)_ = 0.99, p = 0.34, n = 13, S3 Results). Similarly, the observed reductions in WHC for fillets stored for 7 days from Experiment E19 of ~ 2% due to crowding was not significant (LRT = 1.81, df = 1, p = 0.18, n = 26, S4 Results). Mean WHC was 86.6 ± 3.7%. There was no significant correlation between either drip loss or WHC with maximum rigor development time or texture measured on Day 2 or Day 7 (Pearson’s correlation, p > 0.05 in all cases, S1 Results). Furthermore, drip loss and WHC did not correlate significantly (*r* = -0.42, p = 0.15, S1 Results).

### Texture

**Fig 4:**
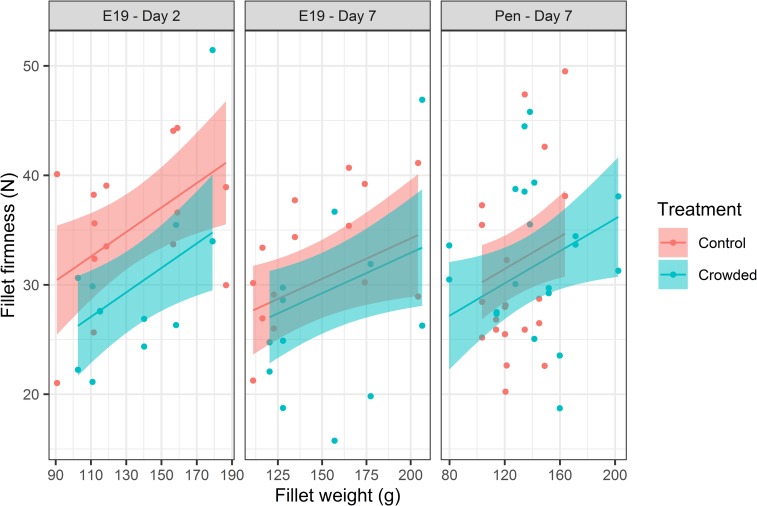
Post-mortem fillet firmness response to crowding in Atlantic mackerel. Fillet firmness were determined at two (“Day 2”) or seven (“Day 7”) days after death and from either Experiment E19 (“E19”) or the net pen experiment (“Pen”). The lines indicate the fitted relationship between fillet weight and firmness, with the 95% confidence intervals shown as shaded areas. The underlying dataset is shown as filled circles. Data points and lines are coloured according to treatment.

Fillet texture was examined at Day 2 and Day 7 for the E19 experiment (n = 52) but only on Day 7 for the net pen experiment (n = 40). At Day 2, the effect of crowding on firmness was independent of fillet weight (LRT = 2.09, df = 1, p = 0.15), but caused a significant reduction (LRT = 4.75, df = 1, p = 0.03) of 15% as a main effect (Fig 4). For the Day 7 model, the interaction between treatment and experiment was marginally significant (interaction model, LRT = 3.98, df = 1, p = 0.05) but treatment was non-significant as a main effect (LRT = 0.46, df = 1, p = 0.49), with texture values for crowded and control fillets being similar (Fig 4). Both models indicated that larger fillets tended to be significantly firmer (LRT, p < 0.05 in both cases). Considering predicted values from the two models for the different days, the mean reduction in fillet texture for the E19 fillets from Day 2 to 7 was 13% for control fillets and 1% for crowded fillets (Fig 4). Fillet firmness did not correlate significantly with initial muscle pH or maximum rigor development time for either Day 2 or Day 7 (Spearman's rank correlation: p > 0.05 in all cases, S1 Results).

### Cathepsin B & L activity

**Fig 5:**
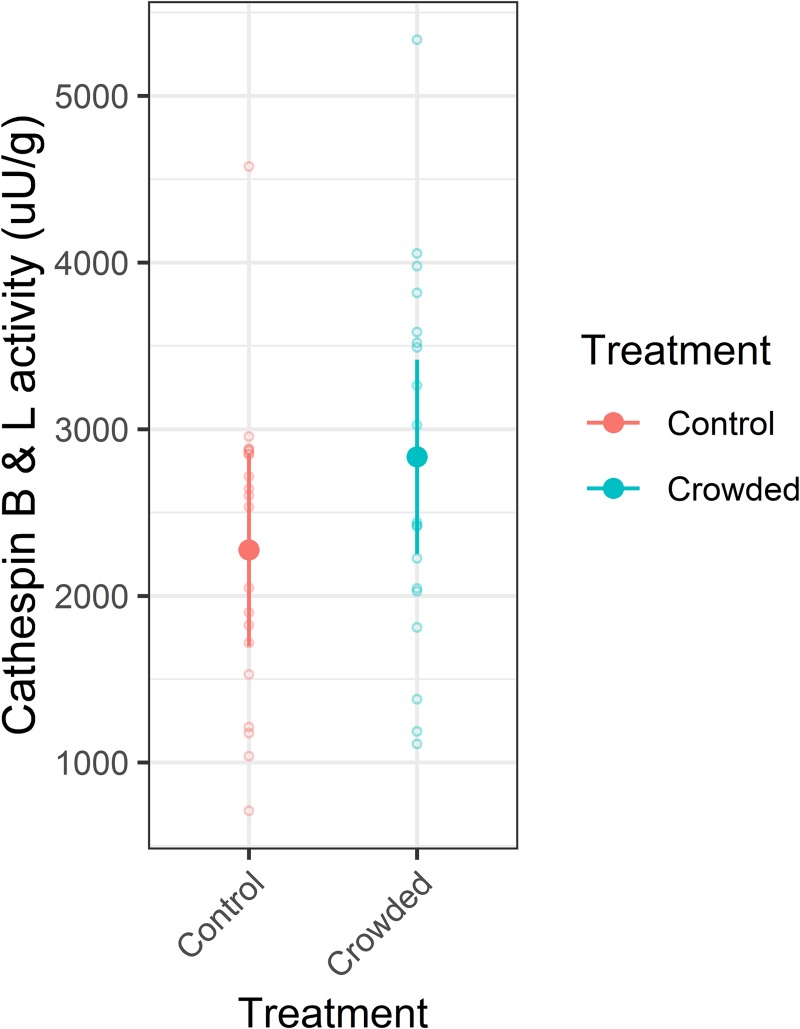
Cathepsin B & L activity response to crowding in Atlantic mackerel. Cathepsin activity was determined seven days after death for fish from the net pen experiment. Model predicted mean values are shown as filled circles, with associated 95% confidence intervals as whiskers. The underlying dataset is shown as open circles. Data points are coloured according to treatment.

We examined cathepsin activity at Day 7 for fish from the net-pen experiment only. There was a tendency for crowded fish to have higher cathepsin B & L activity (an increase of 25%) than the control group (Fig 5), but this difference was not statistically significant (LRT = 1.88, df = 1, p = 0.17, n = 19). Cathepsin activity was not significantly correlated with either fillet firmness at Day 7 (Spearman's rank correlation: ρ = 0.34, p = 0.15) or initial muscle pH (ρ = -0.15, p = 0.51) (S1 Results).

## Discussion

The presented data suggest that crowding of Atlantic mackerel causes a physiological stress response which can have various consequences for subsequent flesh quality. Several other studies on the quality effects of stress have been undertaken in Japan on other members of the *Scomber* genus, including spotted mackerel (*S*. *australasicus*) [40,41] and chub mackerel (*S*. *japonicus*) [42–45]. In accordance with our results, these studies also found a reduction in textural firmness, increased incidence of gaping, lower muscular pH and faster rigor development. These changes were also correlated to pre-mortem physiological stress response observations by some authors [40,41,45]. However, our results are the first to demonstrate these effects in Atlantic mackerel as well as showing fillet colour can be influenced by stress in this genus.

We (and other authors [65,66]) encountered issues with maintaining mackerel in good condition in tanks. Cortisol levels, condition factor and incidence rate of skin injuries suggest that the tank fish were chronically stressed prior to treatment. This is supported by other blood physiology parameters, with elevated ALT (indicative of generalised tissue damage [67]) and reduced triglyceride levels (indicative of longer term mobilisation and subsequent depletion of energy reserves [68]) in the tank fish. It may also have been that by reducing the water volume to allow the efficient collection of fish from the tanks that we applied some degree of “crowding” treatment to the control fish as well; a conjecture that is supported by the slightly elevated lactate control values from the tanks compared to the net pen. The stress response to crowding was however broadly similar to what we observed in the tanks. Our move to the net pen for the final experiment is therefore justifiable, where we were able to obtain better control values of unstressed fish as indicated by the low cortisol values (which are comparable to literature baseline values for Atlantic mackerel [19,69]). Despite the apparent additional stressors and methodological limitations, our tank results can still be considered as valid for demonstrating the comparative effect of crowding treatment on physiology and quality.

The effects of crowding on mackerel blood and muscle physiology are consistent with the current understanding of the acute generalised stress response in teleost fish. This is characterised by increased production and secretion of catecholamines, resulting in glucose release from the liver, liberation of triglycerides into the blood stream as well as increased blood flow to and permeability of gill membranes to allow for increased oxygen uptake [1,70]. Ionic concentration results from osmotic water loss across the now more permeable gills and likely explains the increase in blood ions and osmolality we observed in our mackerel. Although such effects may arise in mackerel from integument damage [65], this is unlikely to be the cause of the differences we observed between crowded and control fish as both groups had similar incidence rates of skin injuries. Cortisol is thought to play a mitigating role against osmotic stress [71] as well perhaps further stimulating glucose generation [72], which accounts for the substantial increases we observed in this hormone. We also observed near significant increases in haematocrit in our mackerel, which can be explained by the combined effects of the observed haemoconcentration and catecholamine activation of the spleen causing an increase in the size and number of circulating erythrocytes to further enhance oxygen availability [70]. Increases in plasma phosphates may reflect hydrolysis of high-energy phosphates due to catecholamine action [73,74], thereby providing energy for increased muscular activity.

The large increases in lactate concentrations as well as the reduction in muscular and plasma pH indicate that the crowding treatment triggered anaerobic metabolism [67,75]. The relatively high levels of dissolved oxygen concentration during crowding suggest this acidosis was primarily metabolic in origin and did not arise due to ambient hypoxia. Pawson and Lockwood [19] and Swift [69] examined mackerel physiology in relation to crowding stress and obtained similar whole blood lactate values to us by collecting control fish with hook and line. However, those authors observed around a two-fold increase in lactate immediately post-crowding, whereas we observed more than a ten-fold increase. This disparity may be accounted for to some degree by differences in physiological response across seasons [76,77], differences in fish size [78] and analytical methods, as well as differences in the applied crowding density and duration. This noted however, our lactate values during crowding are still exceptionally high and far exceed literature values for trawled caught mackerel [19,69,79,80], a capture method that by design involves exhaustive swimming [81]. This would suggest that our crowding treatment was particularly stressful and caused intense physical activity far beyond the aerobic capacity of mackerel [82].

The clear tendency for faster onset and increased rigor strength for the crowded mackerel is consistent with findings from other species (e.g. Roth *et al*. [50]) and other members of the *Scomber* genus [40,43]. These effects arise due to the more rapid depletion of ATP reserves as a result of the pre-mortem stress [3]. The lack of statistical difference over the whole time course may be explained by the high variation in rigor angle between individual fish and relatively small sample size. Despite the large effect size, the lack of statistical difference in the cathepsin analysis may also be similarly explained. In many other studies, rigor development is commonly quantified by calculating the “rigor index” [83]. This method assumes maximum muscle flexibility immediately post-mortem and cannot therefore account for any pre-mortem stiffening effects. By calculating the rigor angle, we were able to show that all crowded mackerel were stiffer than the controls at the point of death. This effect is likely also to be a result of the failure of pre-mortem muscular actomyosin to separate due to ATP depletion in the stressed fish and is a further indication that the crowding treatment we applied was particularly stressful.

The more rapid decline and lower minimum post-mortem muscular pH we observed in crowded fish is also typical, and can be attributed to the increased pre-mortem acidosis for stressed fish being further exacerbated by post-mortem anaerobic metabolism [22]. For crowded cultured Atlantic salmon (*Salmo salar*), post-mortem pH reached 6.7 in ~ 24 hours [50], for cultured sea bass (*Dicentrarchus labrax* [84]) and gilthead seabream (*Sparus aurata* [85]) pH values of around 6.4 were reached in a similar time frame. Within the *Scomber* genus, post-mortem pH values tend to be more acidic; chub mackerel [86] and spotted mackerel muscle pH [40] reached < 6 while Huss [22] noted that large, (presumably) Atlantic mackerel achieve pH values of 5.8–6–0, which is similar to the values we observed. The relatively large anaerobic capacity of the white muscle of *Scombridae* family [87] may account for these lower pH values. The presence of red muscle (and therefore high glycogen content and potential for lactate production) throughout the white muscle of Atlantic mackerel [88] may contribute to this effect.

These post-mortem pH and rigor strength changes are the likely key determinants of the resulting flesh quality effects we observed [22]. Increased rigor strength leads to the tearing of the relatively weak connective tissue between myotomes [89], resulting in increased gaping. Gaping may be further exacerbated by acidic muscular pH conditions, which accelerates the autolytic breakdown of proteins [90]. This is supported by the correlations we observed between gaping scores with post-mortem pH and rigor onset speed. An important group of autolytic enzymes are the cathepsins, which are liberated from the lysosome when cells die and display increases in activity in acidic conditions [25,54]. Protein denaturation and rigor strength in turn contribute to muscle softening [5,44,91] and reductions in muscular WHC [31,32]. The reflective properties of the fillet and thereby colour can in turn be altered by the activity of autolytic enzymes and WHC changes [27,31,92]. Exercise stress is also known to increase the blood flow to muscles [93] which may also contribute to fillet colour changes via the presence of residual blood [94]. The quality changes of increased gaping, colour change and reduction in firmness coupled with increases in cathepsin B & L activity for our crowded mackerel are consistent with these processes, but further research is required to determine the exact causal mechanisms. The reduction in greenness we observed is indicative of a shift towards more red colouration, which would add support a residual blood explanation while the lack of significant response in WHC/drip loss would suggest that water loss was not the primary cause of these effects. The high fat content of Atlantic mackerel muscle may result in a naturally low water content for this species [22,95], making any changes due to stress difficult to detect.

The magnitude of the responses we observed were often dependant on experiment and (although we couldn’t directly test for it due to collinearity issues) the number of storage days. The likely presence of chronic stress in tanks and its absence in the net pen would account for most of the physiological response differences we observed between experiments, while increases in autolytic activity in control fish over time [22] would explain why gaping scores and fillet firmness tended to converge by Day 7. However, for fillet colour, we observed a relatively large reduction in greenness compared to control levels in Experiment E19 that was not as evident in Experiment E20 or the net pen. This difference is difficult to account for, especially considering the similarity between the two tank experiments and the fact that the effect persisted to Day 7. Further work should therefore examine the effect of stress on mackerel fillet colour in detail to determine if the response we saw is reproducible.

Our experimental setup precluded the use of independent control groups, meaning that we cannot completely exclude the possibility that factors other than our treatment were responsible for the physiological and quality responses we saw. However, the fact that we observed stress and quality responses which were broadly consistent with the response of other small pelagic species [16–18,40–44,96] and the lack of other probable explanations would suggest the differences we saw between the pre- and post-crowding groups could be attributed to the treatment we applied. The similar effects throughout the three experiments (which were conducted in two types of experimental arenas, either tanks and net pens, six weeks apart) and the fact we measured pre- and post-crowding groups immediately after one another further supports this. Future experiments should however employ independent control groups to improve the internal validity of results [97]. Furthermore, physiological responses to stress (and any concomitant quality effects [98]) are known to vary with time [76,77] and we conducted our experiments during a relatively short time period during the winter. It would therefore be useful to determine if and how the responses we observed change temporally. This may of be particular relevance with mackerel, which display significant variations in condition and quality throughout the year as result of the accumulation and exhaustion of fat reserves [99,100] and which are targeted by the commercial fleet mainly in autumn. It is worth noting the collection of fish using hook and line has the potential to select for less satiated fish or certain behavioural types [101], which have been shown to be associated with modified physiological responses [102,103]. With our current experimental setup, we were unable to determine if and how this may have influenced our observations but any effects are likely to be minor considering the gregarious nature and intense feeding behaviour of mackerel in schools. Furthermore, the use of hook and line was unavoidable in the absence of other suitable fish collection methods. It would have been optimal to ensure that feeding status was the same between fish from the different experiments, but the net pen experiment was not planned *a priori*. Access to natural sources of feed in the pen may have modified the physiological response of these fish but the effect is likely to be marginal compared to the magnitude of stressor induced effect [104,105].

Fish from Experiment E19 were used to assess rigor development using the Cutting’s tail drop method [50]. The assessment of rigor in this way has been shown to have the potential to influence the quality parameter of gaping due to mechanical breaking of connective tissue [106]. This may account to some degree for the higher gaping scores obtained for fish from Experiment E19 in comparison to E20 for the Day 2 gaping scores. However, as we applied the same method to determine rigor in both the control and crowded groups in E19, our results are still a valid comparative demonstration that crowding stress increases the incidence of gaping.

Finally, it is unclear if and how the magnitude of the physiological and quality responses we observed would be modified during commercial purse seine mackerel catches due to the extreme differences in scale. The likely presence of different and/or additional interacting stressors in catches at sea may further modify or negate any effects. For instance, significant and rapid reductions in ambient hypoxia resulting from densely crowded large catches can be expected at the end of the commercial capture process [16]. Commercial catches are also typically stored in RSW tanks, while we stored our mackerel on ice [107]. Our results are therefore best considered as a demonstration of the potential for crowding stress to influence quality parameters and further work would be required within the fishery to establish if the results are generalisable. Arguably, the responses we observed in the net pen experiment would be less than a wild caught population due to the effects of habituation (as noted by [14,15]). Accordingly, future work should set out to determine any physiological and quality effects from commercial catches. This may be facilitated by the use of validated POC devices (as shown in S1 Validation) to gather indications of relative stress in real time in the field [108].

## Supporting information

S1 Table Measured responses and assessment times for the three different mackerel crowding experiments. Different responses were measured at different time points between the different experiments due to logistic and transport issues. Furthermore, fish from Experiment E20 were used in a separate freezing storage experiment past Day 2 (not reported here).
(PDF)

S1 Validation Validation of point of care (POC) devices. Validation of the Lactate Pro 2 and Contour Next One point of care (POC) devices for the measurement of Atlantic mackerel (*Scomber scombrus*) blood lactate and glucose values.
(DOCX)

S1 Results Spearman and Pearson correlation plots and results. Correlation plots to examine the relationship between a variety of pre-mortem physiological metrics and post-mortem flesh quality variables in Atlantic mackerel.
(DOCX)

S2 Results Post-mortem blood spotting response to crowding in Atlantic mackerel fillets. Blood spotting scores were obtained either two (“Day 2”) or seven (“Day 7”) days after death. Model predicted mean values are shown as filled circles for the different experiments, with associated 95% confidence intervals as whiskers. Data points are coloured according to treatment.
(TIF)

S3 Results Post-mortem drip loss response to crowding in Atlantic mackerel fillets. Drip loss was examined from Experiment E19 only. Model predicted mean values are shown as filled circles, with associated 95% confidence intervals as whiskers. The underlying dataset is shown as open circles. Data points are coloured according to treatment.
(TIF)

S4 Results Post-mortem water holding capacity (WHC) response to crowding in Atlantic mackerel fillets. Water holding capacity was examined from Experiment E19 only. Model predicted mean values are shown as filled circles, with associated 95% confidence intervals as whiskers. The underlying dataset is shown as open circles. Data points are coloured according to treatment.
(TIF)

S1 Dataset Biometric dataset. (CSV)

S2 Dataset Blood spotting scores dataset. (CSV)

S3 Dataset Cathepsin B & L activity dataset. (CSV)

S4 Dataset Post-mortem flesh and skin colour dataset. (CSV)

S5 Dataset Drip loss dataset. (CSV)

S6 Dataset Gaping scores dataset. (CSV)

S7 Dataset Pre-mortem physiology dataset. (CSV)

S8 Dataset Post-mortem rigor and muscle pH dataset. (CSV)

S9 Dataset Fillet firmness dataset. (CSV)

S10 Dataset Water holding capacity dataset. (CSV)
