# Supplementary material for: Physiological and flesh quality consequences of pre-mortem crowding stress in Atlantic mackerel (Scomber scombrus)
Source: PLoS One. 2020 Feb 13;15(2):e0228454. doi: 10.1371/journal.pone.0228454 (PMC7018012; doi:10.1371/journal.pone.0228454)
Supplement: S1 Table — Different responses were measured at different time points between the different experiments due to logistic and transport issues. Furthermore, fish from Experiment E20 were used in a separate freezing storage experiment past Day 2 (not reported here). (PDF) [file pone.0228454.s001.pdf]

Storage	Sampling time	Experiment		
		E19	E20	Net pen
**Fresh**	**0 hour**	- Blood physiology	- Blood physiology	- Blood physiology
		- Muscle pH	- Muscle pH	- Muscle pH
**Ice stored**	**≤ 43 hours**	- Rigor angle		
		- Muscle pH (post-mortem)		
	**Day 2**	- Colour (flesh)	- Colour (flesh)	
		- Colour (skin)	- Colour (skin)	
		- Blood spotting	- Blood spotting	
		- Gaping	- Gaping	
		- Texture		
		- Fillet weight (drip loss)		
	**Day 7**	- Colour (flesh)		- Colour (flesh)
		- Blood spotting		- Colour (skin)
		- Gaping		- Blood spotting
		- Texture		- Gaping
		- Water holding capacity		- Texture
		- Fillet weight (drip loss)		
**Frozen (-80°C)**				Cathepsins B & L*

* fillets were frozen after 7 days of ice storage and later defrosted for cathepsin analysis.
