# Supplementary material for: Physiological and flesh quality consequences of pre-mortem crowding stress in Atlantic mackerel (Scomber scombrus)
Source: PLoS One. 2020 Feb 13;15(2):e0228454. doi: 10.1371/journal.pone.0228454 (PMC7018012; doi:10.1371/journal.pone.0228454)
Supplement: S1 Validation — Validation of the Lactate Pro 2 and Contour Next One point of care (POC) devices for the measurement of Atlantic mackerel (Scomber scombrus) blood lactate and glucose values. (DOCX) [file pone.0228454.s002.docx]

**S1 Validation – Validation of the Lactate Pro 2 and Contour Next One point of care (POC) devices for the measurement of Atlantic mackerel (*Scomber scombrus*) blood lactate and glucose values.**

*Methodology*

We regressed Atlantic mackerel (*Scomber scombrus*) whole blood lactate and glucose values obtained by the point of care (POC) Lactate Pro 2 and Contour Next One devices respectively against defrosted plasma values obtained in the laboratory, from either the ABL90-FLEX blood gas analyser or the MAXMAT PL biomedical analyser. We assumed that results obtained from the laboratory-based devices offered a better estimate of the true value. Please refer to the main article for detail of experimental procedures as well as sample preparation and storage. Selected specifications for the different devices used are included in Table A.

Standard linear regression of whole blood against plasma values indicated heterogenetic residuals. We therefore incorporated a fixed variance structure into the models (top left of each panel in Figure A), to allow for a larger residual spread with increasing whole blood values [1]. We also fitted separate ANCOVA style models to determine whether the fitted regression line was significantly different from the expected 1:1 relationship between whole blood and plasma values. Spearman’s rho (ρ) was used as a non-parametric measure of correlation between whole blood and plasma values. We visually examined plots of Cook’s distance [2] to determine influential/outlier values for each regression.

**Table A: Lactate and glucose device specifications.** Selected specifications of the Lactate Pro 2, Contour Next One and ABL-90 FLEX devices used to determine blood lactate and glucose values in Atlantic mackerel.

| **Device** | Lactate Pro 2 | Contour Next One | ABL-90 FLEX | MAXMAT PL |
| --- | --- | --- | --- | --- |
| **Company** | Arkray Inc. | Ascensia | Radiometer Medical | MaxMat SA |
| **Type** | Point of care device | Point of care device | Blood gas analyser | Biomedical analyser |
| **Parameters tested** | Whole blood lactate | Whole blood glucose | Plasma lactate  Plasma glucose | Plasma lactate |
| **Additional test kit required** | Yes | Yes | No | Yes |
| **Required sample amount (µL)** | 0.3 | 0.6 | 65 | 150 |
| **Method of measurement** | Lactate oxidase enzyme electrode method | FAD-GDH enzyme electrode method | Amperometry | Spectrophotometry |
| **Range (mmol/L)** | 0.5 – 25 | 1.1 – 33 | Lactate: 0 - 31  Glucose: 0 – 60 | 0.1 – 13.2 |

*Results*

The correlation between whole blood and plasma lactate values was strongly positive (Spearman’s ρ = 0.87, p < 0.001), with no significance difference (F = 0.87, df = 1, p = 0.35) from the expected 1:1 relationship. The relationship was particularly strong at lower lactate levels (which tended to come from the control samples) but tended to be weaker for crowded fish, as shown by higher degree of scatter in the data for higher lactate values (Fig. A, left panel). Cook’s distance plots suggested at least two observations (4% of the dataset) could be considered outliers, indicating particularly poor agreement between the POC device and laboratory analysis for these values (indicated on Fig. A, left panel by *).

For glucose, the correlation between the POC and laboratory device values was also strongly positive (Spearman’s ρ = 0.81, p < 0.001). However, the whole blood values obtained from the POC device substantially underestimated the plasma values (Fig. A, right panel). This difference was significant (F = 295.87, df = 1, p < 0.001) and based on the current dataset, a mean (±SE) adjustment of +1.78 ± 0.10 mmol/L on values obtained by the Contour Next One would be required to estimate plasma glucose levels. At least three observations (6% of the dataset) could be considered as outliers (indicated on Fig. A, right panel by *).

*Conclusions*

The results indicate that, in field conditions which preclude the storage of plasma samples, the Lactate Pro 2 and Contour Next One may be used as informative methods of determining relative changes in lactate and glucose levels in Atlantic mackerel. However, determination of absolute values using these devices should currently be avoided due to the occasional large discrepancy between whole blood and laboratory plasma values (especially for higher lactate values) as well as the consistent underestimation of plasma glucose values. Further collection of data over a wider range of glucose values would allow for the calculation of a more accurate adjustment factor on the POC derived values.

*References*

1. Zuur A, Ieno EN, Walker N, Saveliev AA, Smith GM. Mixed Effects Models and Extensions in Ecology with R. New York: Springer-Verlag; 2009.

2. Fox J. Regression Diagnostics: An Introduction. SAGE; 1991.


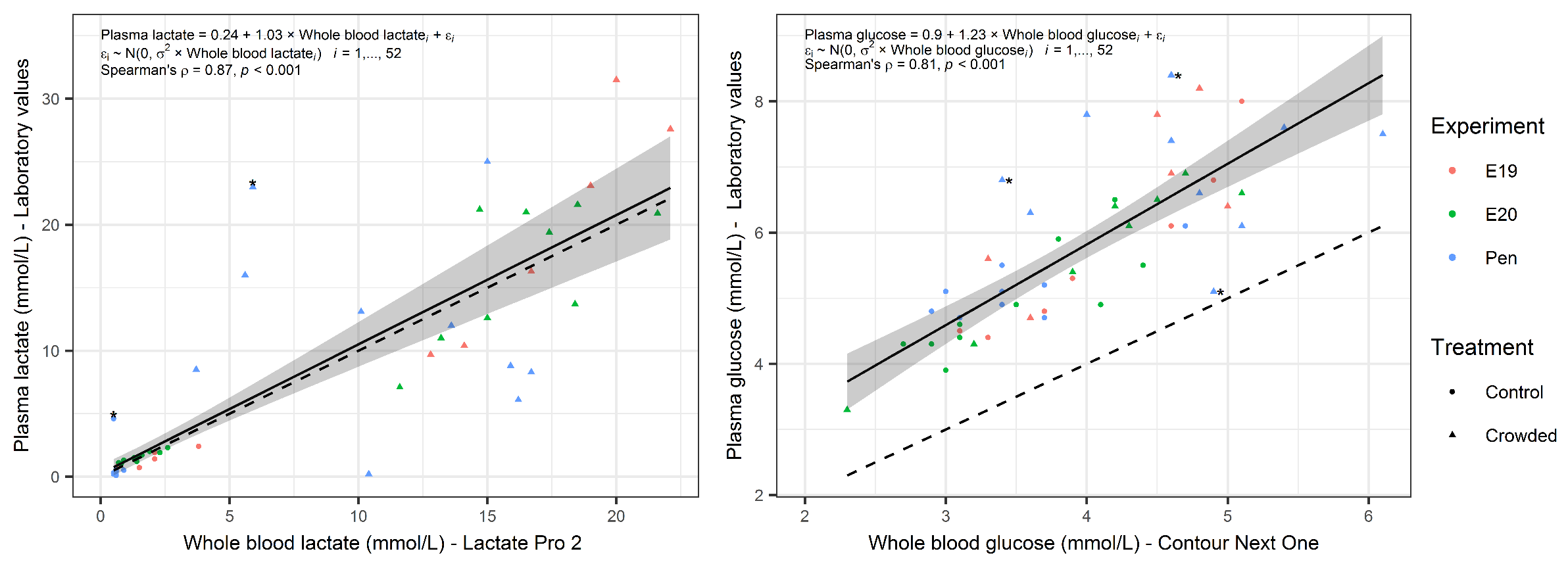


**Fig A: Regression analysis for Atlantic mackerel blood lactate and glucose values.** Whole blood values were analysed immediately following extraction using either the Lactate Pro 2 (left panel) or Contour Next One (right panel) point of care devices. Plasma values were obtained from defrosted plasma samples using a ABL-90 FLEX laboratory based blood gas analyser. The solid line indicates the fitted relationship (detailed further in top left of each plot), with the 95% confidence intervals shown as the shaded area. The dashed line shows the expected relationship (1:1). The underlying data set is coloured according to experiment. Outlier observations as suggested by Cook’s distance analysis are marked by *.
