# Supplementary material for: Physiological and flesh quality consequences of pre-mortem crowding stress in Atlantic mackerel (Scomber scombrus)
Source: PLoS One. 2020 Feb 13;15(2):e0228454. doi: 10.1371/journal.pone.0228454 (PMC7018012; doi:10.1371/journal.pone.0228454)
Supplement: S1 Results — Correlation plots to examine the relationship between a variety of pre-mortem physiological metrics and post-mortem flesh quality variables in Atlantic mackerel. (DOCX) [file pone.0228454.s003.docx]

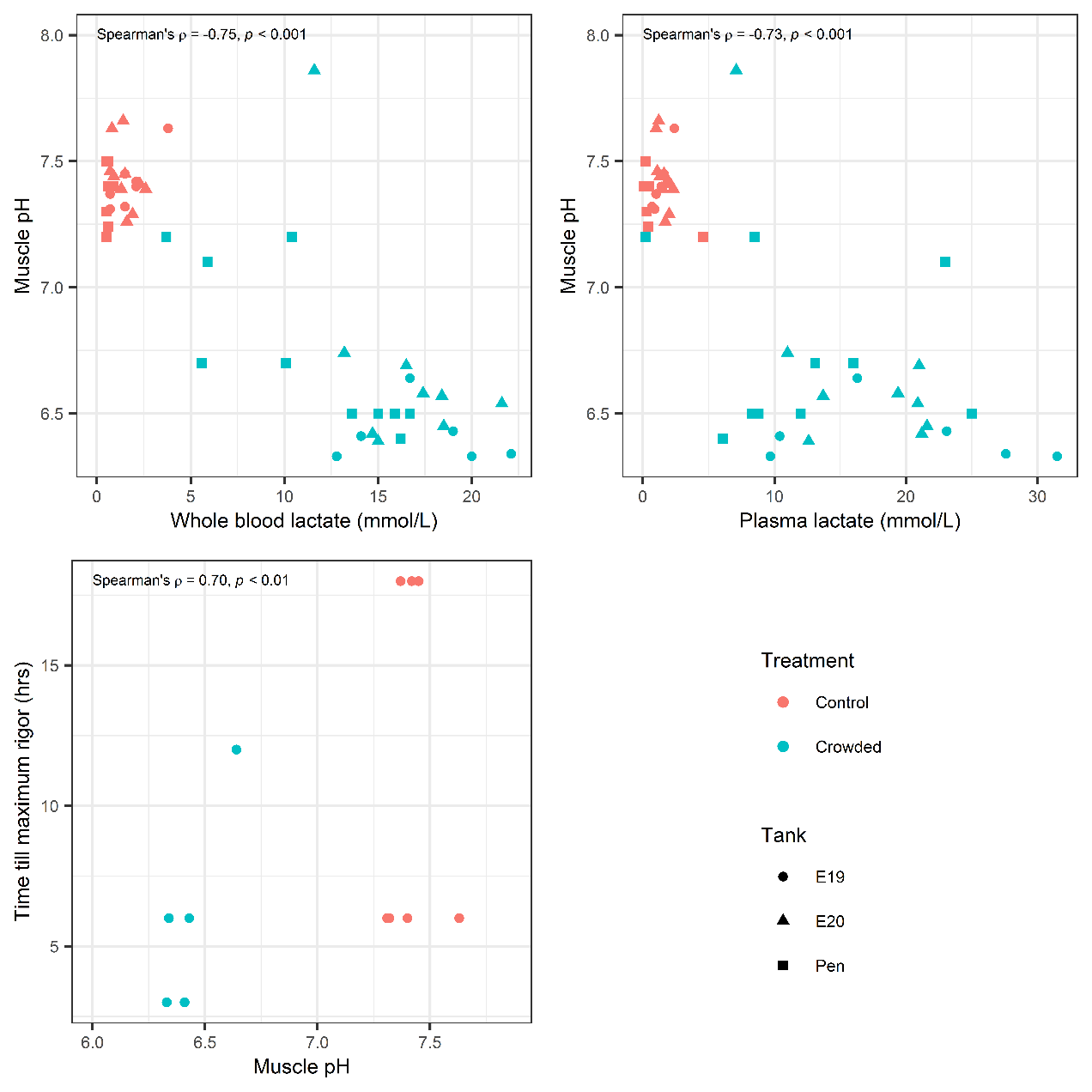


**Fig A:** Correlations between various pre-mortem physiological variables and time till maximum post-mortem rigor in Atlantic mackerel. Please refer to the main article for detail of experimental design.


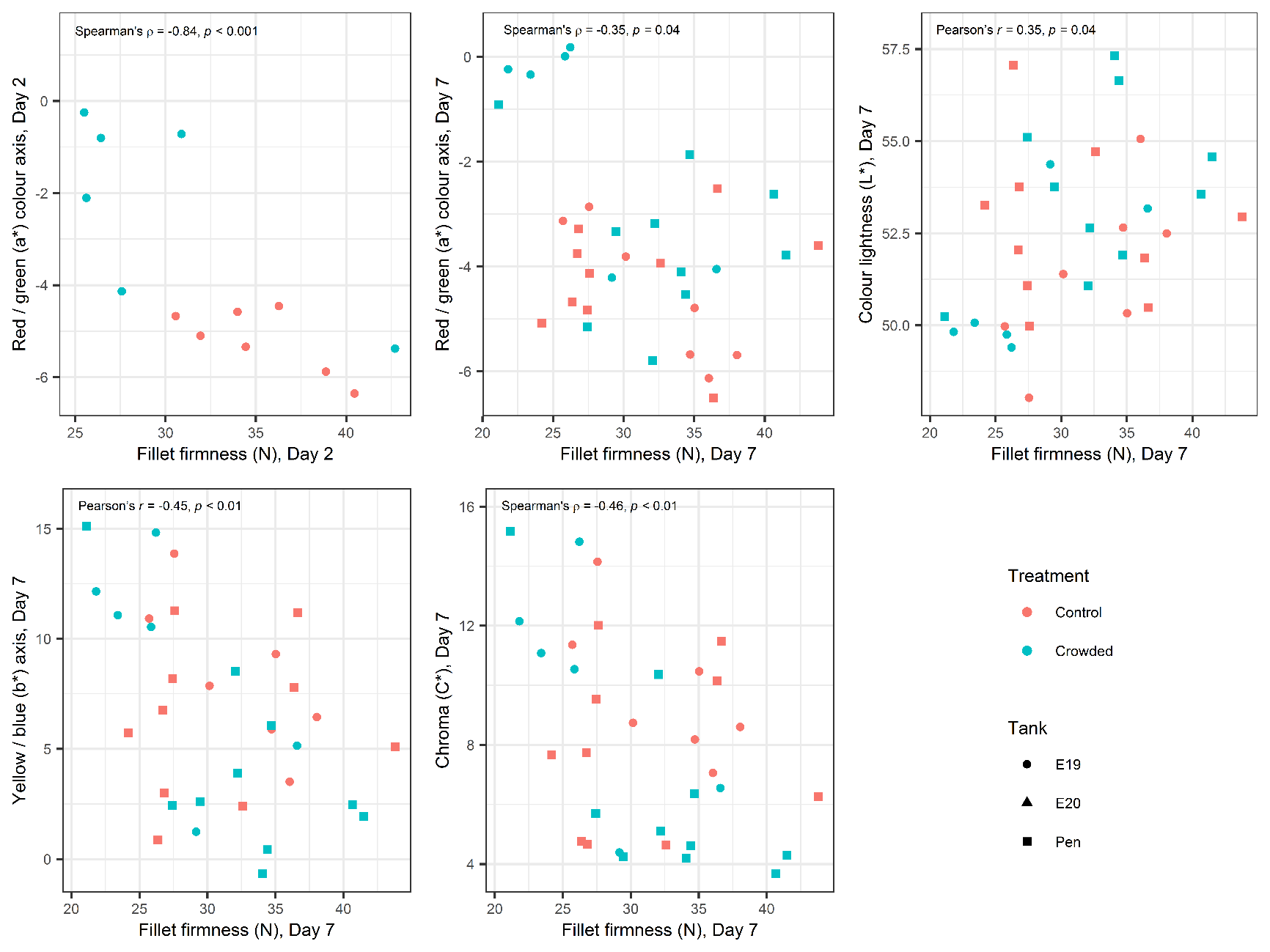


**Fig B:** Correlations between post-mortem flesh colour variables and fillet firmness in Atlantic mackerel. Please refer to the main article for detail of experimental design.
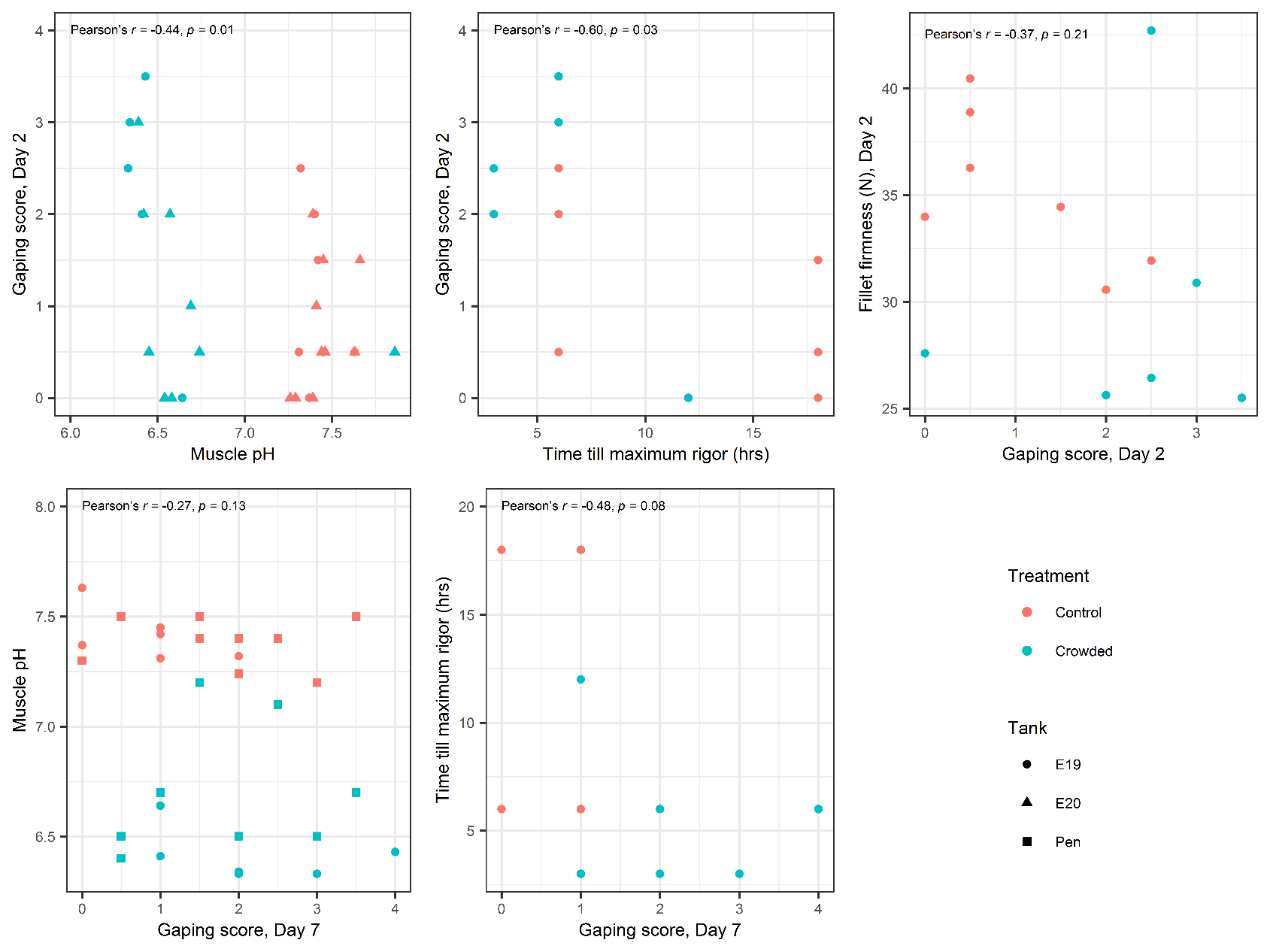


**Fig C:** Correlations between post-mortem gaping scores and various flesh quality variables in Atlantic mackerel. Please refer to the main article for detail of experimental design.


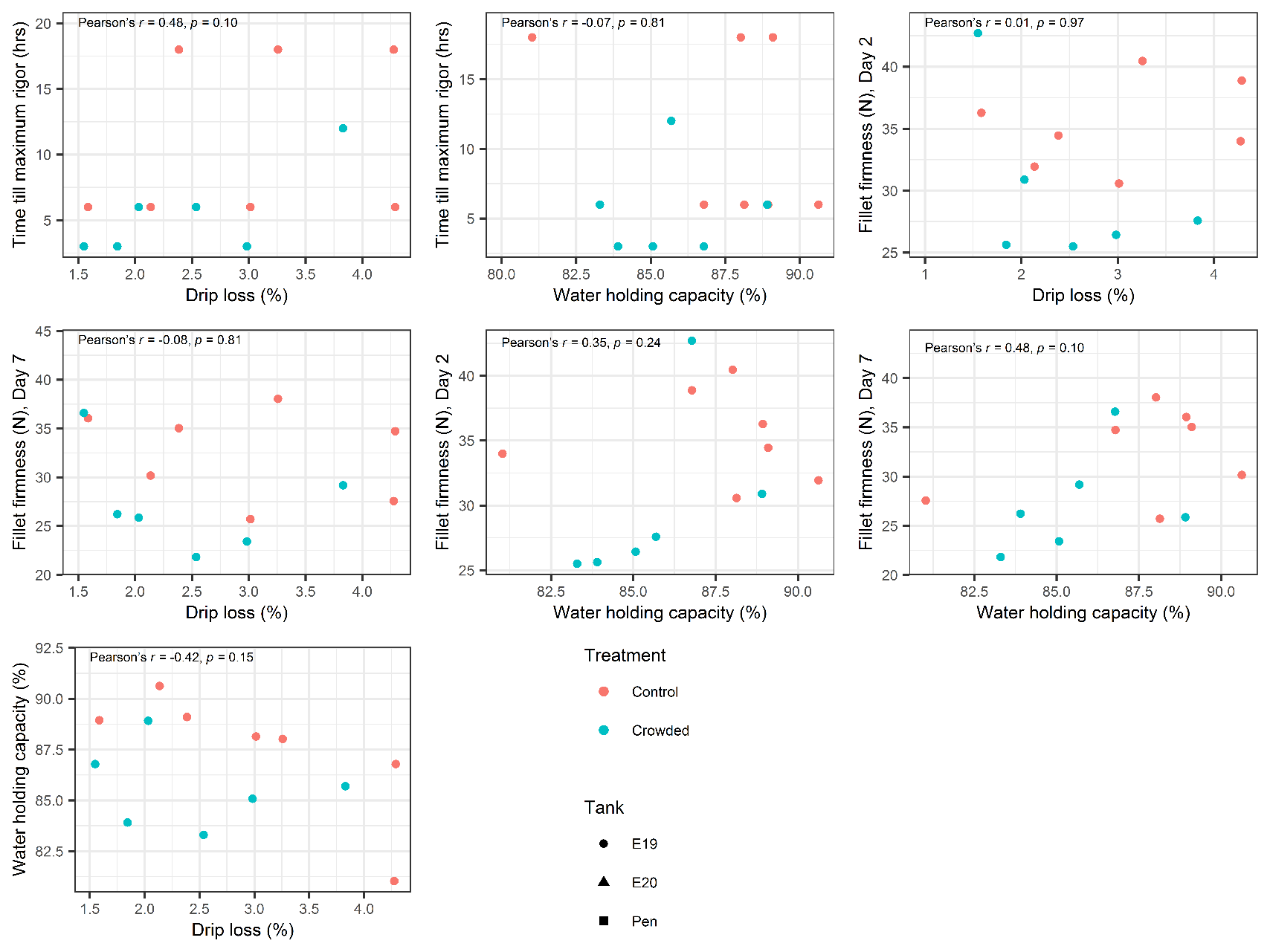


**Fig D:** Correlations between post-mortem water holding capacity, drip loss and various flesh quality variables in Atlantic mackerel. Please refer to the main article for detail of experimental design.


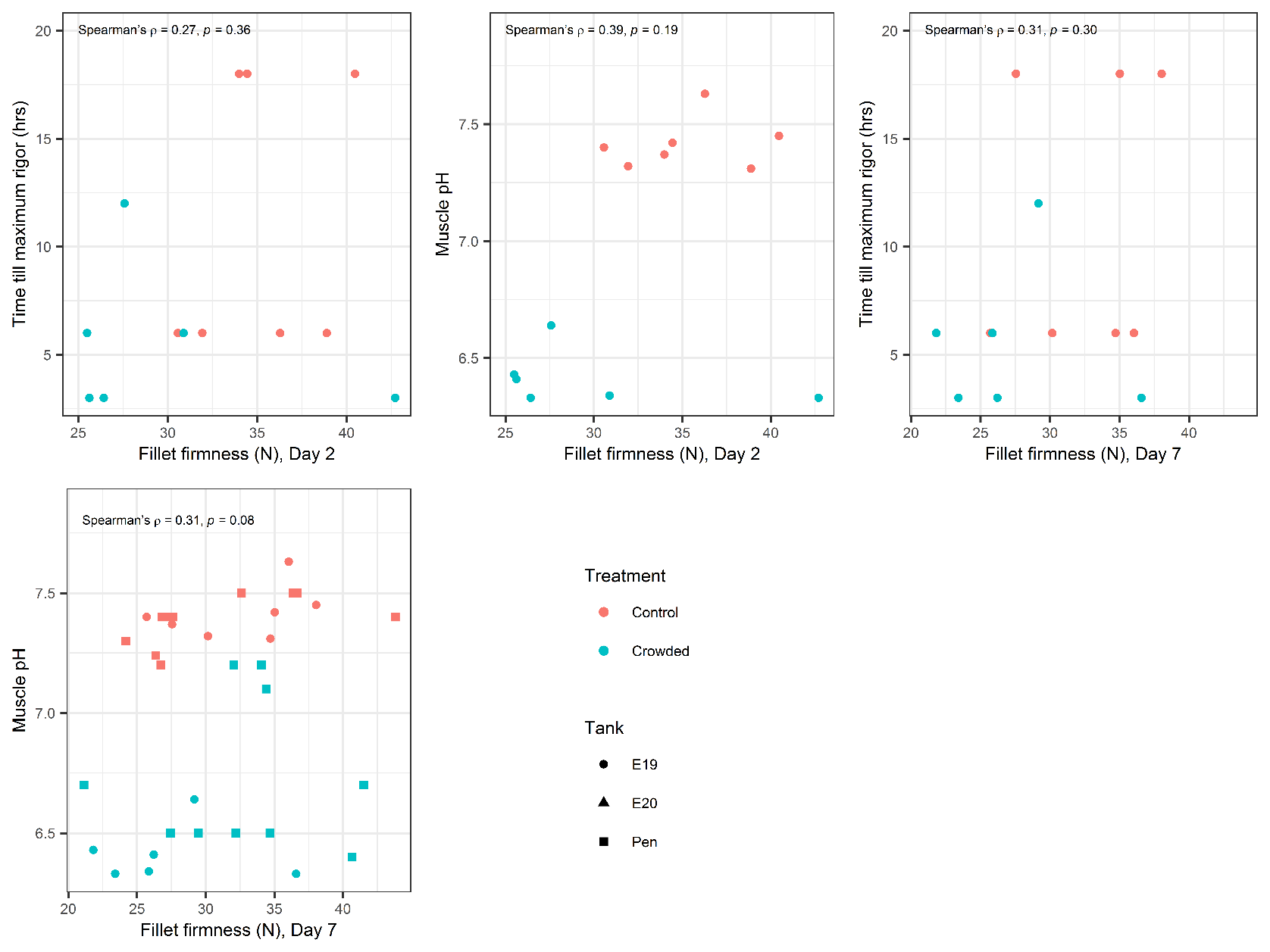


**Fig E:** Correlations between post-mortem fillet firmness and various flesh quality variables in Atlantic mackerel. Please refer to the main article for detail of experimental design.


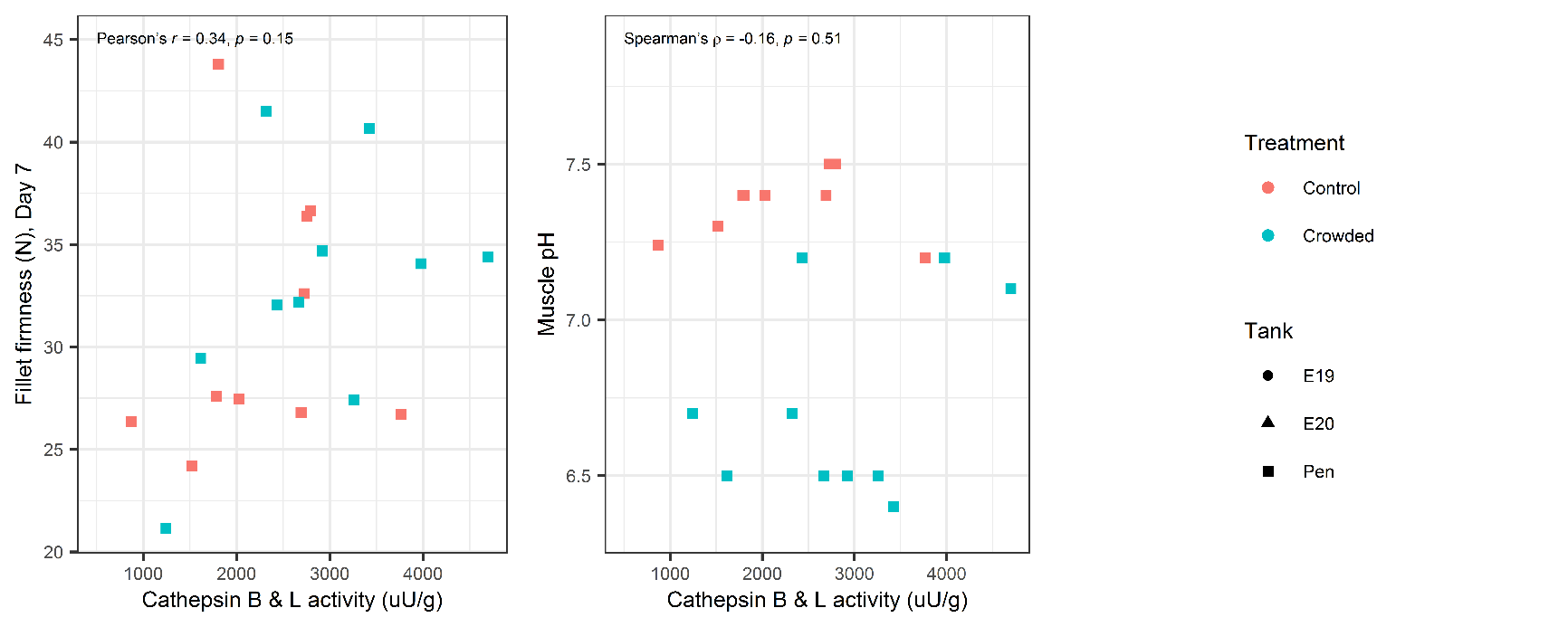


**Fig F:** Correlations between post-mortem cathepsin B & L activity and fillet firmness or muscle pH in Atlantic mackerel. Please refer to the main article for detail of experimental design.
