# Supplementary figures and images for: Physiological and flesh quality consequences of pre-mortem crowding stress in Atlantic mackerel (Scomber scombrus)
Source: PLoS One. 2020 Feb 13;15(2):e0228454. doi: 10.1371/journal.pone.0228454 (PMC7018012; doi:10.1371/journal.pone.0228454)

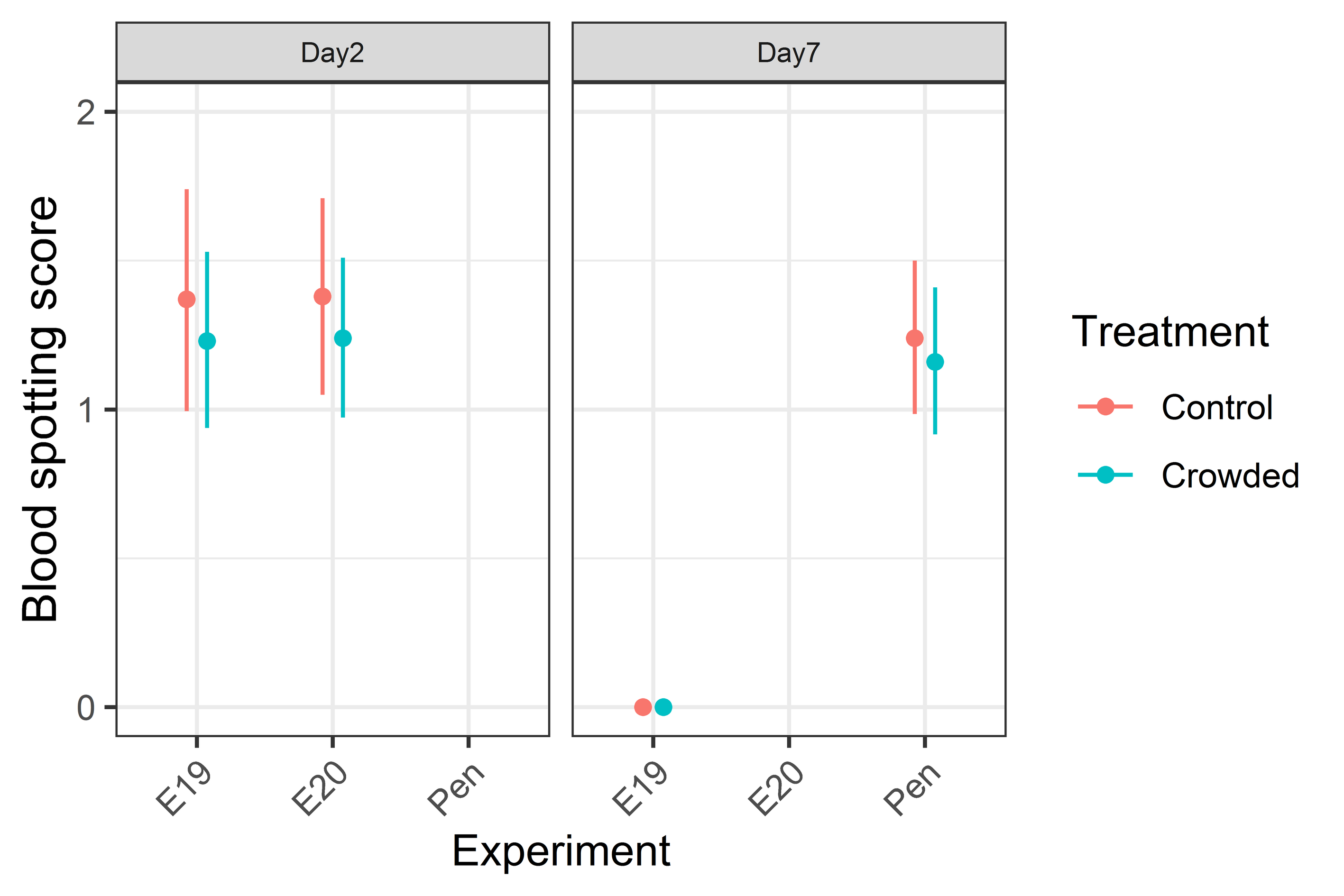

Supplement: S2 Results — Blood spotting scores were obtained either two (“Day 2”) or seven (“Day 7”) days after death. Model predicted mean values are shown as filled circles for the different experiments, with associated 95% confidence intervals as whiskers. Data points are coloured according to treatment. (TIF) [file pone.0228454.s004.tif]

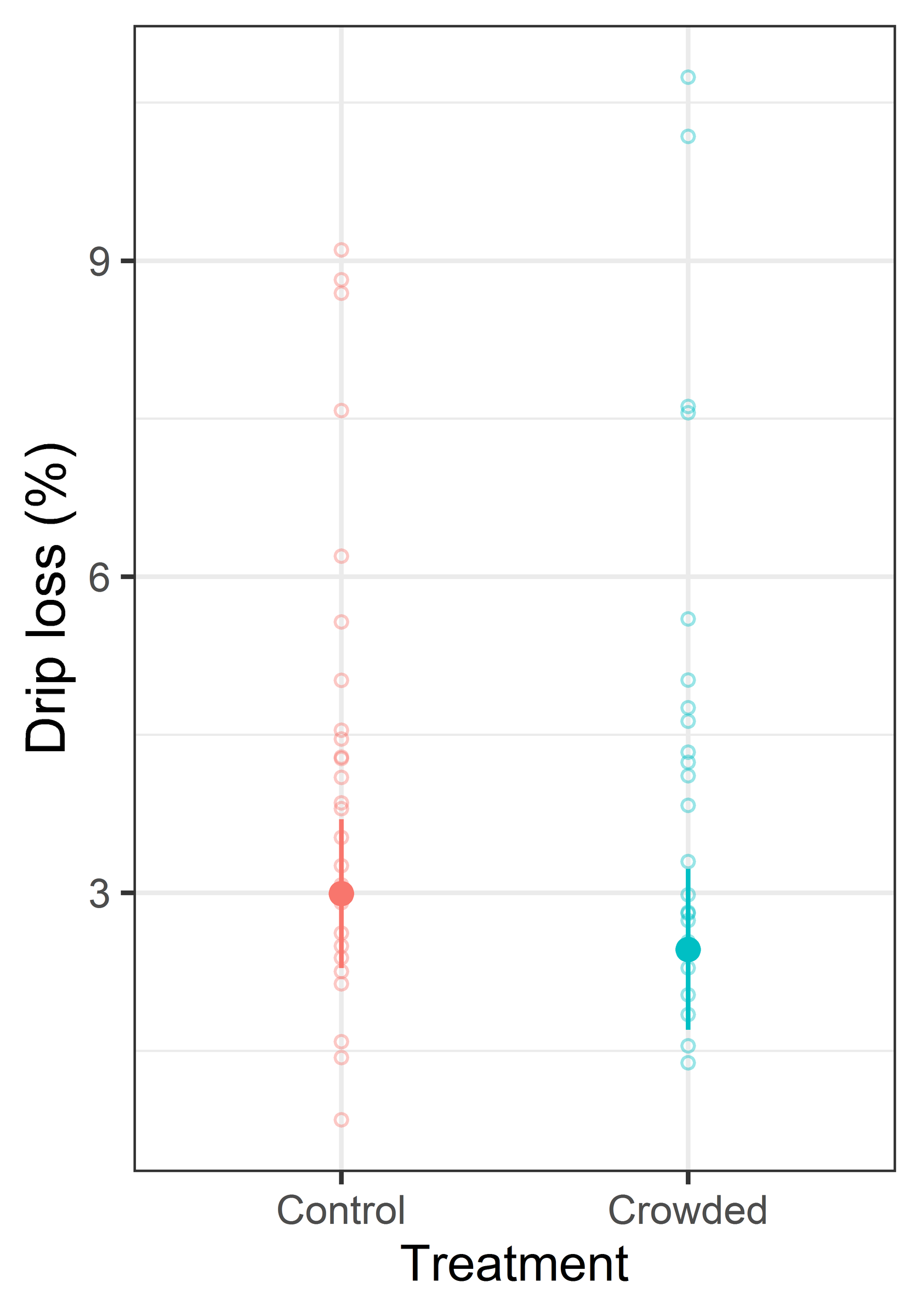

Supplement: S3 Results — Drip loss was examined from Experiment E19 only. Model predicted mean values are shown as filled circles, with associated 95% confidence intervals as whiskers. The underlying dataset is shown as open circles. Data points are coloured according to treatment. (TIF) [file pone.0228454.s005.tif]

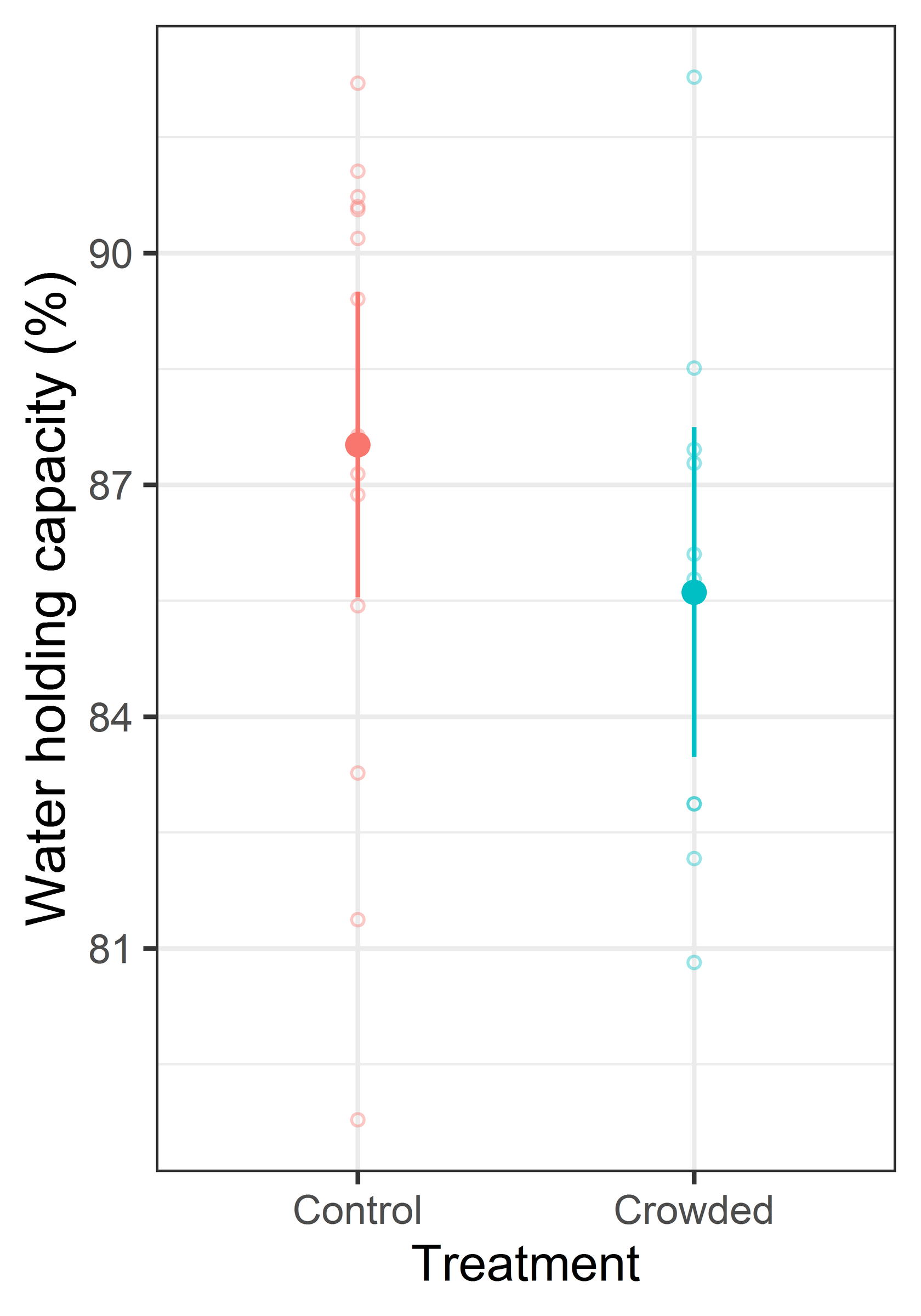

Supplement: S4 Results — Water holding capacity was examined from Experiment E19 only. Model predicted mean values are shown as filled circles, with associated 95% confidence intervals as whiskers. The underlying dataset is shown as open circles. Data points are coloured according to treatment. (TIF) [file pone.0228454.s006.tif]
